# DHA Targets RAB1B to Mediate the Driving Force Transmission of Copper Ion Vesicular Endocytosis Under Copper Overload for Promoting Cuproptosis and Ferroptosis in Hepatic Stellate Cells

**DOI:** 10.7150/ijbs.134520

**Published:** 2026-07-22

**Authors:** Xinran Qiu, Haoyuan Tian, Shujiang Sun, Ningyu Qiu, Biao Xu, Junrui Wang, Yuanyuan Gao, Zhengyang Bao, Xiang Han, Jie Zeng, Yuxin Lin, Feng Zhang, Shizhong Zheng, Jiangjuan Shao

**Affiliations:** 1 Jiangsu Key Laboratory for Pharmacology and Safety Research of Chinese Materia Media, Nanjing University of Chinese Medicine, Nanjing 210023, China.; 2 College of Electronic and Optical Engineering & College of Flexible Electronics (Future Technology), Nanjing University of Posts and Telecommunications, Nanjing 210023, China.; 3 State Key Laboratory of Flexible Electronics (LoFE) & Institute of Advanced Materials (IAM), School of Materials Science and Engineering, Nanjing University of Posts & Telecommunications, 9 Wenyuan Road, Nanjing, 210023 China.

**Keywords:** liver fibrosis, cuproptosis, dihydroartemisinin, ferroptosis, vesicular endocytosis, biomimetic nanoliposomes

## Abstract

Liver fibrosis represents a central pathological process in chronic liver diseases and poses a severe threat to human health. Cuproptosis, a copper ion-dependent form of regulated cell death, offers a potential therapeutic strategy for liver fibrosis. This study demonstrated that dihydroartemisinin (DHA) transiently upregulates SLC31A1, thereby promoting copper influx. Under copper overload, SLC31A1 undergoes ubiquitin-mediated degradation, while DHA targets RAB1B to activate RAB10-dependent vesicular endocytosis as a compensatory mechanism for sustained copper accumulation. This endocytic process alters cellular biomechanical properties and strengthens RAB1B-VDAC1 interaction to promote mitochondrial copper uptake. Following cuproptosis, iron-sulfur cluster proteins undergo degradation, leading to massive iron release and subsequent ferroptosis, establishing a cascading dual cell death mechanism. Mechanistically, RAB1B regulates KAT2A-mediated succinylation of USP11, thereby inhibiting USP11's deubiquitinating activity and accelerating SLC31A1 degradation. For *in vivo* applications, we engineered biomimetic nanoliposomes coated with HSC membranes and surface-functionalized with CD47, FNIII10, and ApoE. This system integrates immune evasion, hepatic accumulation, and HSC-specific recognition capabilities, demonstrating remarkable targeting efficacy and mechanistic consistency in primary HSCs and rodent models. These findings reveal that DHA enhances copper-laden vesicular endocytosis by targeting RAB1B, thereby triggering cuproptosis and ferroptosis, and provide novel molecular targeting strategies for liver fibrosis therapy.

## 1. Introduction

Liver fibrosis has become a globally increasing health concern, posing a severe threat to human health^1^. Liver fibrosis is characterized by hepatic stellate cell (HSC) activation, excessive extracellular matrix (ECM) deposition, and accompanying hepatocyte injury, inflammation, and destruction of hepatic lobular architecture, it may progress to cirrhosis or hepatocellular carcinoma^2^. Effective therapeutic strategies are currently limited in clinical practice; therefore, elucidating the core pathological mechanisms is essential to provide a theoretical basis for effective treatment.

Copper and iron are essential elements in liver metabolism, playing critical roles in various physiological processes^3^. The liver serves as the central hub for copper and iron metabolism, regulating the levels of these metal ions to maintain homeostasis^4^, ^5^. Dysregulation of copper and iron metabolism may contribute to hepatic injury and fibrosis^6^. Recent studies have revealed that copper overload not only damages cells through traditional oxidative stress mechanisms but may also induce a novel form of cell death known as cuproptosis^7^, ^8^. Unlike apoptosis or necrosis, cuproptosis is a copper-dependent form of cell death characterized by the lipoylation of mitochondrial proteins^9^. Ferroptosis, a form of iron-dependent cell death^10^, may potentially interact with cuproptosis^11^, ^12^. Excessive copper accumulation impairs mitochondrial function and exacerbates oxidative stress, thereby disrupting iron metabolism and promoting iron accumulation within the cell^13^. This process represents a key mechanism driving ferroptosis^14^. Our findings suggest that during cuproptosis, excessive copper ions interact with iron-sulfur cluster proteins, disrupting their normal function. This leads to the excessive accumulation of iron within the cell, exacerbating lipid peroxidation and driving ferroptosis.

When cells face copper and iron ion imbalance, they initiate multi-layered homeostatic regulation to maintain metal ion homeostasis^15^. Cells mitigate adverse effects and protect themselves from damage by regulating the uptake, storage, and transport of metal ions^16^. Cells may utilize metal-binding proteins, such as copper-zinc superoxide dismutase (Cu/Zn-SOD) or ferritin, to sequester excess copper and iron ions, decreasing their free ion state and minimizing the oxidative reactions triggered by these metals^17^. Antioxidant enzymes, such as glutathione peroxidase (GPx) and catalase (CAT), initiate antioxidant defense mechanisms within the cell by decomposing free radicals and peroxides, thereby reducing oxidative stress^17^. Cells may also rely on vesicular transport systems to regulate ion compartmentalization. Vesicles are small structures enclosed by lipid bilayers within the cell^18^. They play a crucial role in intracellular and extracellular material transport, signal transduction, and metabolic regulation through processes such as endocytosis, exocytosis, and vesicle-mediated transport, which are essential for cellular physiological processes^19^. Therefore, vesicles may play a critical role in the regulation of metal ion transport. The directional transport of vesicles typically relies on specific members of the small GTPase family within the cell^20^. The RAB family, as a crucial group of small GTPases, serves as a central regulatory factor in intracellular membrane trafficking^21^. RAB1B, a critical member of the RAB family, plays a pivotal role in the transport of materials between the endoplasmic reticulum and the Golgi apparatus. It is essential in regulating vesicle formation, trafficking, and fusion with target membranes^22^. In addition to molecular regulation, vesicle transport is accompanied by significant biomechanical effects^23^. During vesicular transport, the cell membrane undergoes bending and deformation, leading to changes in membrane tension^24^. These changes in tension cause certain proteins to be exposed or aggregated, further regulating vesicle formation and fusion with target membranes^23^. Moreover, the interaction patterns of relevant membrane proteins change when the cell experiences mechanical force, causing some proteins to transition from an inactive to an active state, thereby providing the foundation for mechano-biochemical signal coupling^25^.

To further explore the mechanisms of cell death triggered by metal ion accumulation, we focused on a potential therapeutic agent DHA. DHA not only exhibits significant antimalarial activity but also shows promising potential in the treatment of liver fibrosis^26^-^28^. Our previous studies have demonstrated that DHA alleviates liver fibrosis by inducing ferroptosis in HSCs, a process regulated by m^6^A methylation^29^. DHA, through its unique peroxide bridge structure, interacts with metal ions (such as copper and iron), disrupting the homeostasis of intracellular metal ions and promoting their accumulation^30^. This effect leads to increased production of reactive oxygen species (ROS), which subsequently depletes antioxidant molecules, such as glutathione (GSH)^31^. GSH stabilizes metal ions by binding to them, thereby regulating metal ion homeostasis and mitigating oxidative stress and cellular damage induced by metal overload^32^. Therefore, the downregulation of GSH not only directly diminishes the cell's antioxidant defense capacity but also facilitates the accumulation of copper and iron, thereby activating cell death mechanisms such as cuproptosis and ferroptosis. This provides a potential link between DHA-induced metal ion accumulation and the activation of these cell death pathways. Despite the promising therapeutic potential of DHA, its clinical application is hindered by low bioavailability. In recent years, various novel nanodrug delivery systems (NDDS) have been continuously designed and optimized, demonstrating strong therapeutic potential while significantly reducing the side effects commonly associated with traditional drug treatments^33^-^35^. Among these, cell membrane-coated biomimetic nanoparticles have shown great promise in targeted therapy due to their excellent targeting ability, immune evasion capabilities, and biocompatibility^36^, ^37^. To further enhance therapeutic efficacy, we designed and constructed biomimetic nanoliposomes coated with HSC membranes and functionalized their surface with three protein molecules: CD47, FNIII10, and ApoE. This system not only inhibits phagocytosis but also enhances targeting to HSCs.

In summary, this study revealed cuproptosis characteristics of HSCs during hepatic fibrosis progression via single-cell sequencing. DHA activates cuproptosis through RAB1B-mediated regulation of copper ion uptake, subsequently triggering ferroptosis. Specifically, DHA upregulates SLC31A1 at early stages to promote sustained copper influx; under copper overload, DHA targets RAB1B to regulate RAB10-mediated vesicular endocytosis of copper ions. This endocytic process alters the biomechanical properties of HSCs, enhances the interaction between RAB1B and VDAC1, and facilitates copper ion influx into mitochondria. Cuproptosis-mediated loss of iron-sulfur cluster proteins secondarily induces ferroptosis, forming a cascading cell death mechanism. Meanwhile, RAB1B regulates KAT2A-induced succinylation of USP11, inhibiting its deubiquitinating activity and enhancing SLC31A1 ubiquitination levels, thereby strengthening vesicular endocytosis and accelerating cell death. These findings establish RAB1B as a critical regulator of the cuproptosis-ferroptosis cascade and provide novel therapeutic targets. Furthermore, HSC membrane-coated biomimetic nanoliposomes improved DHA bioavailability and targeted delivery, offering new perspectives for hepatic fibrosis treatment.

## 2. Results

### 2.1 Single-Cell RNA Sequencing Unveils the Role of Cuproptosis in the Pathogenesis of Liver Fibrosis

**Figure 1:**
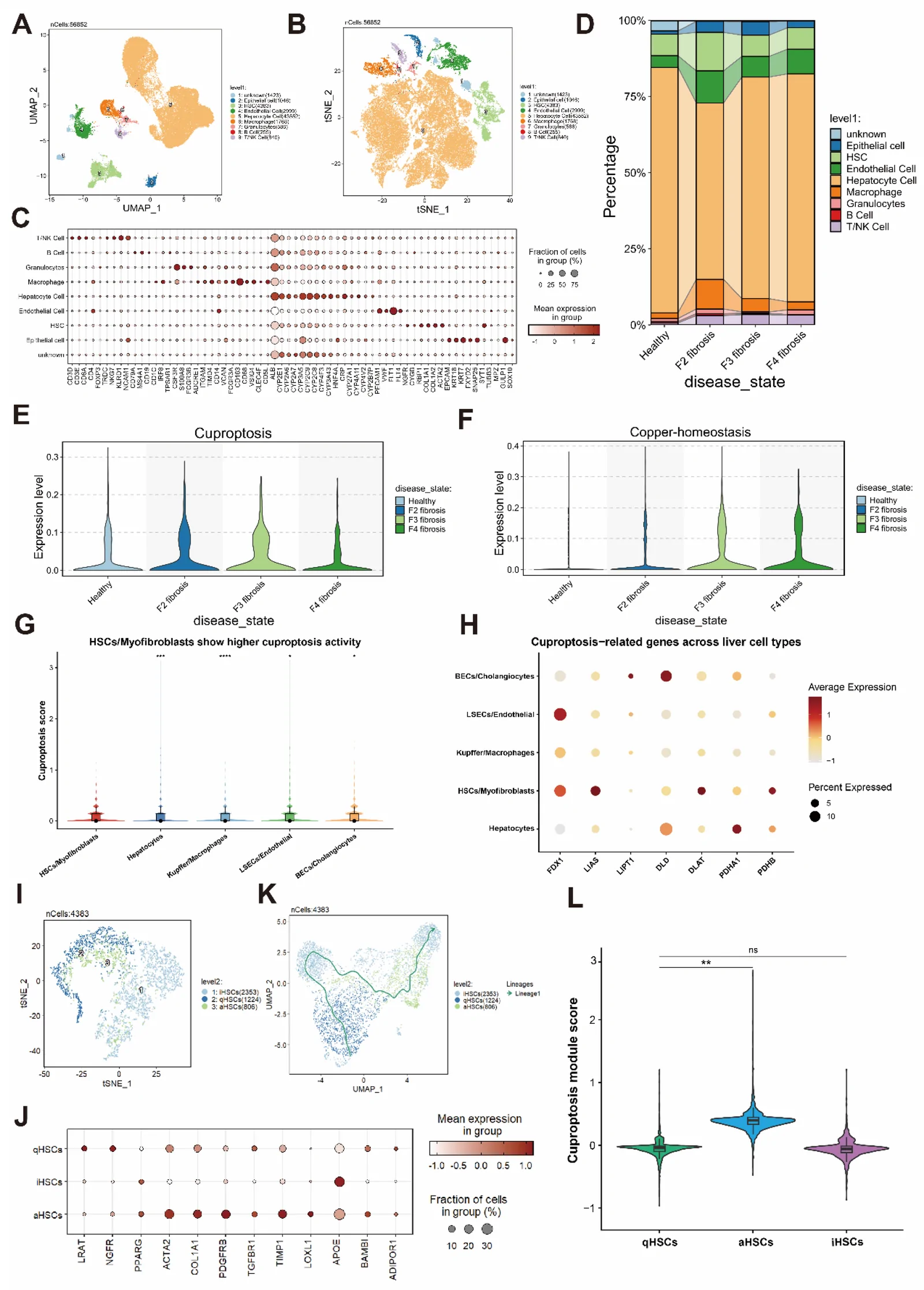
** Single-cell RNA sequencing unveils the role of cuproptosis in the pathogenesis of liver fibrosis.** (A-B) Uniform Manifold Approximation and Projection (UMAP) and t-Distributed Stochastic Neighbor Embedding (t-SNE) analyses of 56,852 single cells, with clustering annotated by canonical markers. (C) UMAP visualization showing the expression levels of selected markers across all cell subtypes. The size of the dots represents the proportion of expressing cells, while the color reflects the normalized gene expression levels. (D) Variation in the proportion of annotated cell types across different groups. (E) Changes in cuproptosis scores across different stages of liver fibrosis (F0-F4). (F) Changes in copper homeostasis scores across different stages of liver fibrosis (F0-F4). (G) Cuproptosis scores among five distinct cell types, including hepatocytes, endothelial cells, Kupffer cells, HSCs, and cholangiocytes. (H) Expression patterns of cuproptosis-related genes across the five cell types. Dot size indicates the proportion of expressing cells, and color intensity denotes normalized expression levels. (I) Re-clustering of HSCs and annotation of subpopulations. (J) Expression profiles of canonical markers defining the three HSCs subpopulations. Dot size indicates the proportion of expressing cells, and color intensity denotes normalized expression levels. (K) Pseudotime analysis of HSCs subpopulations. (L) Cuproptosis gene module scores in qHSCs, aHSCs, and iHSCs. Data are presented as mean ± SD, with p-values calculated using one-way ANOVA. ns, not significant; *p < 0.05, **p < 0.01.

Cuproptosis, a newly identified form of programmed cell death in recent years, is characterized by the direct binding of copper ions to lipoylated proteins in the TCA cycle, inducing their aberrant oligomerization and leading to the destabilization of iron-sulfur cluster proteins^9^. To investigate the cell-type specificity and molecular characteristics of cuproptosis during liver fibrosis progression, this study retrieved publicly available single-cell RNA sequencing (scRNA-seq) data (GSE210077) from the Gene Expression Omnibus (GEO) database and systematically analyzed liver tissues from healthy controls and subjects with varying degrees of fibrosis. Following quality control, batch effect correction, and dimensionality reduction clustering, a total of 62,542 high-quality cells were retained and annotated into nine major cell types, including epithelial cells, HSCs, endothelial cells, hepatocytes, macrophages, granulocytes, B cells, T/NK cells, and unassigned cells (Figure 1A-C). Inter-sample analysis of cell proportions revealed that the proportions of HSCs and endothelial cells progressively increased as liver fibrosis advanced, whereas the proportion of hepatocytes decreased concomitantly. These findings indicate that HSCs activation constitutes a central mechanism driving liver fibrosis, consistent with established pathological hallmarks of this disease^38^ (Figure 1D).

Focusing on cuproptosis-related gene sets, we observed that overall cuproptosis scores and copper homeostasis-related gene expression progressively increased during liver fibrosis progression, indicating significant copper metabolic dysregulation and cuproptosis activation at advanced fibrotic stages (Figure 1E-F). Further comparison across cell clusters showed that the cuproptosis score in HSCs/myofibroblast populations was markedly higher than that in hepatocytes, endothelial cells, Kupffer cells, and other parenchymal and immune cells. Additionally, cuproptosis driver genes (e.g., FDX1 and LIAS) exhibited the highest average expression in HSCs (Figure 1G-H). These results demonstrate that HSCs activation represents a pivotal cellular event for cuproptosis during liver fibrosis progression. To validate this dynamic relationship *in vitro*, primary mouse HSCs were isolated and treated with TGF-β and PDGF-BB for 0-12 days to simulate the activation process. Time-course analysis revealed that cuproptosis-related gene expression remained at basal levels during early activation, whereas massive HSC activation was accompanied by progressively enhanced cuproptosis and elevated fibrosis marker expression (Supplementary Fig. S1A-C). These findings confirm that HSCs activation is closely associated with cuproptosis progression, and that copper metabolic reprogramming coupled with mitochondrial lipoic acid pathway activation significantly increases susceptibility to cuproptosis. Based on these observations, we hypothesize that targeted induction of cuproptosis in activated HSCs may serve as a potential therapeutic strategy against liver fibrosis.

Given the dynamic activation profile of HSCs during fibrogenesis, the HSC population was subjected to subclustering. Based on the expression of canonical markers, three distinct subpopulations were identified: quiescent HSCs (qHSCs), marked by *Lrat*, *Ngfr*, and *Pparg*; activated HSCs (aHSCs), characterized by *Col1a1*, *Acta2*, *Timp1*, and *Loxl1*; and inactivated/suppressed HSCs (iHSCs), which partially re-expressed quiescent markers including *Pparg*, *Apoe*, and *Bambi* (Figure 1I-J). Following the annotation of HSC subpopulations, we performed pseudotime analysis (Figure 1K). Furthermore, analysis of cuproptosis module scores across HSC subpopulations revealed that qHSCs exhibited basal levels, whereas aHSCs showed significantly elevated scores. In contrast, iHSCs displayed reduced scores relative to aHSCs, returning to levels approaching those of qHSCs. These findings indicate that HSC activation is accompanied by markedly enhanced cuproptosis activity, whereas inactivation partially alleviates cuproptosis, restoring levels to near-quiescent states. This subpopulation-specific difference confirms that cuproptosis activity is closely associated with HSC functional status, and that aHSCs represent the primary effector cell population for cuproptosis (Figure 1L).

In summary, cuproptosis may play a critical role in the development and progression of liver fibrosis. Dysregulation of copper homeostasis may represent an essential pathological mechanism underlying HSC activation and fibrosis progression.

### 2.2 DHA Chronologically Activates Cuproptosis and Ferroptosis to Cascadingly Inhibit HSCs Proliferation

**Figure 2:**
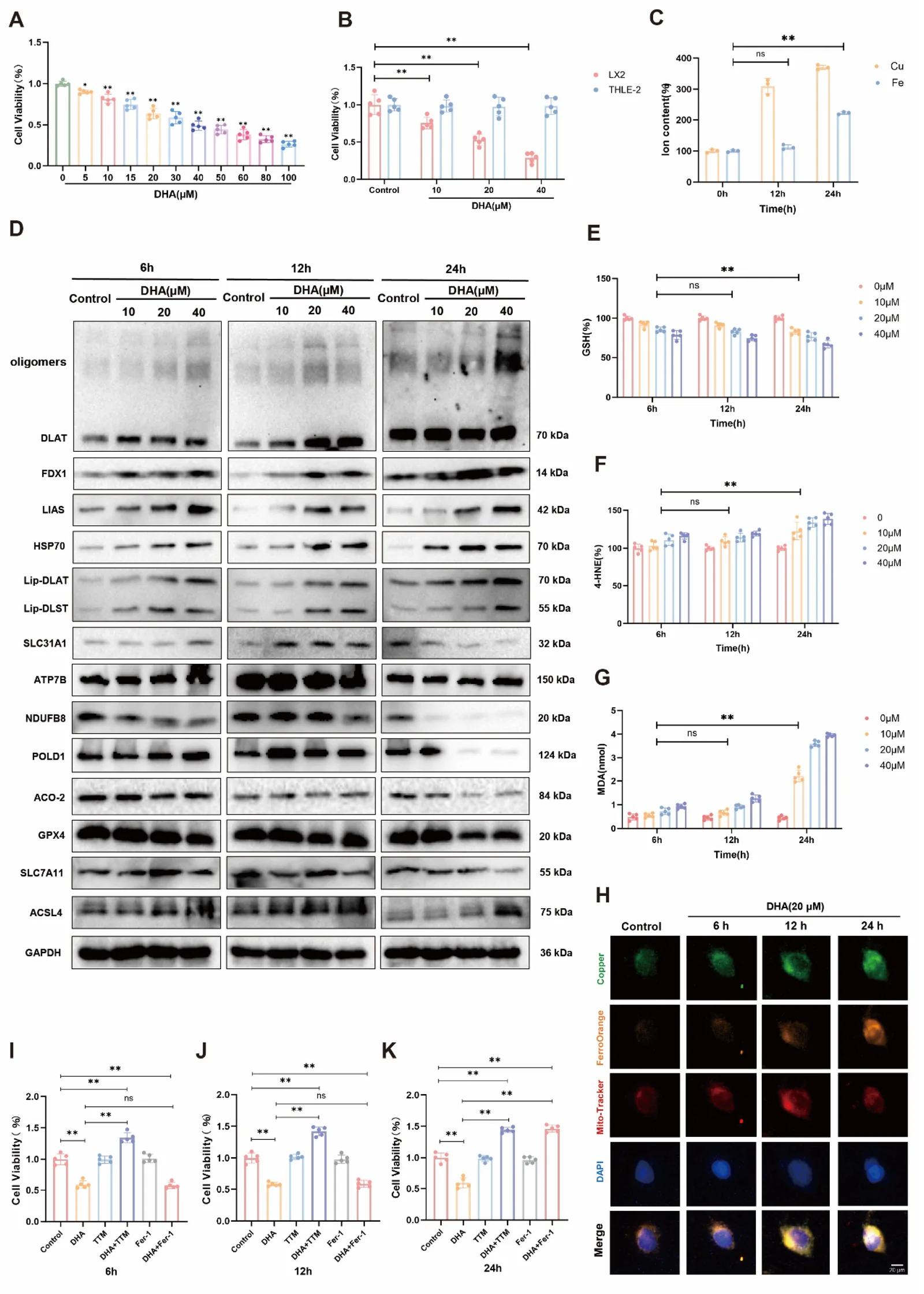
** DHA chronologically activates cuproptosis and ferroptosis to cascadingly inhibit HSC proliferation.** (A) LX2 cells were treated with DHA (0-100 µM) for 24 hours to determine the optimal concentration for DHA intervention (n = 5). (B) LX2 and THLE-2 cells were treated with DHA (0-40 µM) for 24 hours. DHA significantly inhibited the viability of LX2 cells, while having no apparent effect on normal hepatocytes (THLE-2) (n = 5). (C) ICP-MS analysis was performed to measure copper and iron ion concentrations at different time points following DHA (20 µM) treatment (n = 3). (D) Western blot analysis was used to examine the expression levels of proteins involved in the oligomerization of DLAT, FDX1, LIAS, HSP70, Lip-DLAT, Lip-DLST, SLC31A1, ATP7B, GPX4, SLC7A11, ACSL4, NDUFB8, POLD1, ACO-2 in LX2 cells treated with DHA (0-40 µM) for 6 h, 12 h, and 24 h. Protein expression levels were quantified using grayscale analysis (n = 3). (E) LX2 cells were treated with DHA (0-40 µM) for 6 h, 12 h, and 24 h, and the intracellular glutathione levels were measured at each time point using a glutathione assay kit (n = 3). (F) LX2 cells were treated with DHA (0-40 µM) for 6 h, 12 h, and 24 h, and the intracellular 4-HNE levels were measured at each time point using a 4-HNE assay kit (n = 3). (G) LX2 cells were treated with DHA (0-40 µM) for 6 h, 12 h, and 24 h, and the intracellular MDA levels were measured at each time point using an MDA assay kit (n = 3). (H) LX2 cells were treated with DHA (20 µM) for 6 h, 12 h, and 24 h, and the co-localization of copper ions (green), iron ions (orange), and mitochondria (red) was observed by immunofluorescence at different time points (n = 3). Scale bar: 20 µm. (I-K) LX2 cells were treated with DHA (20 µM), TTM (5 µM), or Fer-1 (10 µM), either separately or in combination, for 6 h, 12 h, and 24 h. Changes in cell viability were assessed using the CCK-8 assay (n = 5). Data are presented as mean ± SD, with p-values calculated using one-way ANOVA. ns, not significant; *p < 0.05, **p < 0.01.

Our previous research has demonstrated the significant potential of DHA in the treatment of liver fibrosis. The unique endoperoxide bridge confers metal-binding capacity, while concomitant GSH depletion attenuates antioxidant defenses^30^. To investigate the molecular mechanisms underlying DHA-mediated inhibition of HSC proliferation, we first determined the optimal intervention concentration of DHA in LX2 cells through cell proliferation assays (CCK-8) (Figure 2A). At the same concentration, DHA significantly suppressed LX2 cell viability without exerting notable effects on normal hepatocytes, indicating the potential of DHA to specifically inhibit LX2 proliferation (Figure 2B). ICP-MS analysis revealed differential dynamics of intracellular copper and iron ion contents following DHA intervention: intracellular copper levels significantly increased at the early stage of intervention, whereas iron accumulation occurred at 24 hours (Figure 2C). These findings indicate that DHA may trigger cuproptosis prior to ferroptosis, suggesting a sequential activation of these two distinct cell death modalities.

To determine whether the selective cytotoxicity of DHA against HSCs originates from differential intrinsic susceptibility to cuproptosis, pHeps and pHSCs were isolated. Following DHA treatment, pHSCs exhibited marked upregulation of DLAT oligomers and lipoylated DLAT/DLST, whereas these markers remained unchanged in pHeps. Concomitantly, ferroptosis-related proteins SLC7A11 and GPX4 were downregulated in pHSCs, as were activation markers α-SMA and Collagen I; both profiles remained stable in pHeps. These findings confirm that DHA selectively eliminates HSCs through concurrent activation of cuproptosis and ferroptosis pathways, without exerting significant effects on hepatocytes with higher thresholds for cuproptosis (Supplementary Fig. S2A-B). To further elucidate the mechanism by which DHA activates cuproptosis, we performed gradient concentration interventions at three distinct time points (6 h, 12 h, and 24 h) and analyzed the biomarkers of cuproptosis. The results demonstrated that DHA dose-dependently induced DLAT protein oligomerization and significantly upregulated the expression of key cuproptosis-related proteins, including FDX1, LIAS, and HSP70, at all time points; concurrently, the expression of lipoic acid-modified proteins was also markedly increased (Figure 2D, Supplementary Fig. S2C-E). Notably, the copper efflux protein ATP7B showed no significant changes at any time point, whereas the copper influx protein SLC31A1 was significantly upregulated at the early stages of 6 h and 12 h, suggesting that DHA may enhance intracellular copper accumulation by promoting copper ion influx. At 24 h, SLC31A1 expression was markedly downregulated (Figure 2D, Supplementary Fig. S2C-E).

During cuproptosis, excessive copper ions interact directly with intracellular iron-sulfur cluster proteins, leading to their structural dissociation or inactivation, thereby triggering the release of free iron ions^39^. These iron ions participate in the Fenton reaction to generate free radicals, thereby facilitating ferroptosis initiation^40^, ^41^. To investigate this process, we assessed the expression levels of intracellular iron-sulfur cluster proteins. The results revealed a significant decrease in the expression of iron-sulfur proteins, such as NADH: Ubiquinone Oxidoreductase Subunit B8 (NDUFB8), Polymerase Delta 1 (POLD1), and Aconitase 2 (ACO-2) at 24 h (Figure 2D, Supplementary Fig. S2E). Furthermore, examination of ferroptosis-related biomarkers revealed that SLC7A11, GPX4, and ACSL4 remained unchanged at 6 h and 12 h (Figure 2D, Supplementary Fig. S2C-D). However, at 24 h, SLC7A11 and GPX4 were downregulated in a dose-dependent manner, while ACSL4 showed a marked upregulation (Figure 2D, Supplementary Fig. S2E). As anticipated, the early decrease in GSH levels indicated that cells had initiated a metabolic imbalance associated with cuproptosis (Figure 2E). At 6 h and 12 h, no significant changes in lipid peroxidation products, such as malondialdehyde (MDA) and 4-hydroxynonenal (4-HNE), were observed, indicating that ferroptosis had not yet been activated. However, at 24 h, alongside the continued decline in GSH, significant increases in 4-HNE and MDA were observed, marking the typical features of ferroptosis (Figure 2F-G).

Furthermore, changes in intracellular copper and iron ion levels and their co-localization with mitochondria were examined. Results showed that intracellular copper accumulation progressively increased with prolonged DHA treatment; iron levels increased significantly at 24 h (Figure 2H). Moreover, the co-localization levels of copper and iron ions with mitochondria also increased in a time-dependent manner (Figure 2H, Supplementary Fig. S2F). These findings confirmed that mitochondria represent the convergence point of cuproptosis and ferroptosis. Cell viability rescue assays revealed that the cuproptosis inhibitor significantly enhanced cell viability at all time points (6 h, 12 h, and 24 h). In contrast, the ferroptosis inhibitor only restored cell viability at 24 h, with no significant improvement observed at 6 h and 12 h interventions (Figure 2I-K). Furthermore, various cell death inhibitors were employed to determine whether they could counteract DHA-induced cytotoxicity. Results showed that cuproptosis serves as the core pathway of DHA-induced cell death, whereas ferroptosis acts as a downstream event. In contrast, apoptosis, pyroptosis, and necroptosis were not involved in DHA-mediated suppression of cell viability (Supplementary Fig. S2G).

Chronological analysis revealed that DHA-induced early copper accumulation triggers cuproptosis, subsequently activating ferroptosis pathways at later stages, thereby inhibiting HSC proliferation.

### 2.3 DHA Mediates Mitochondrial Copper Import and Death Signal Amplification through VDAC1

**Figure 3:**
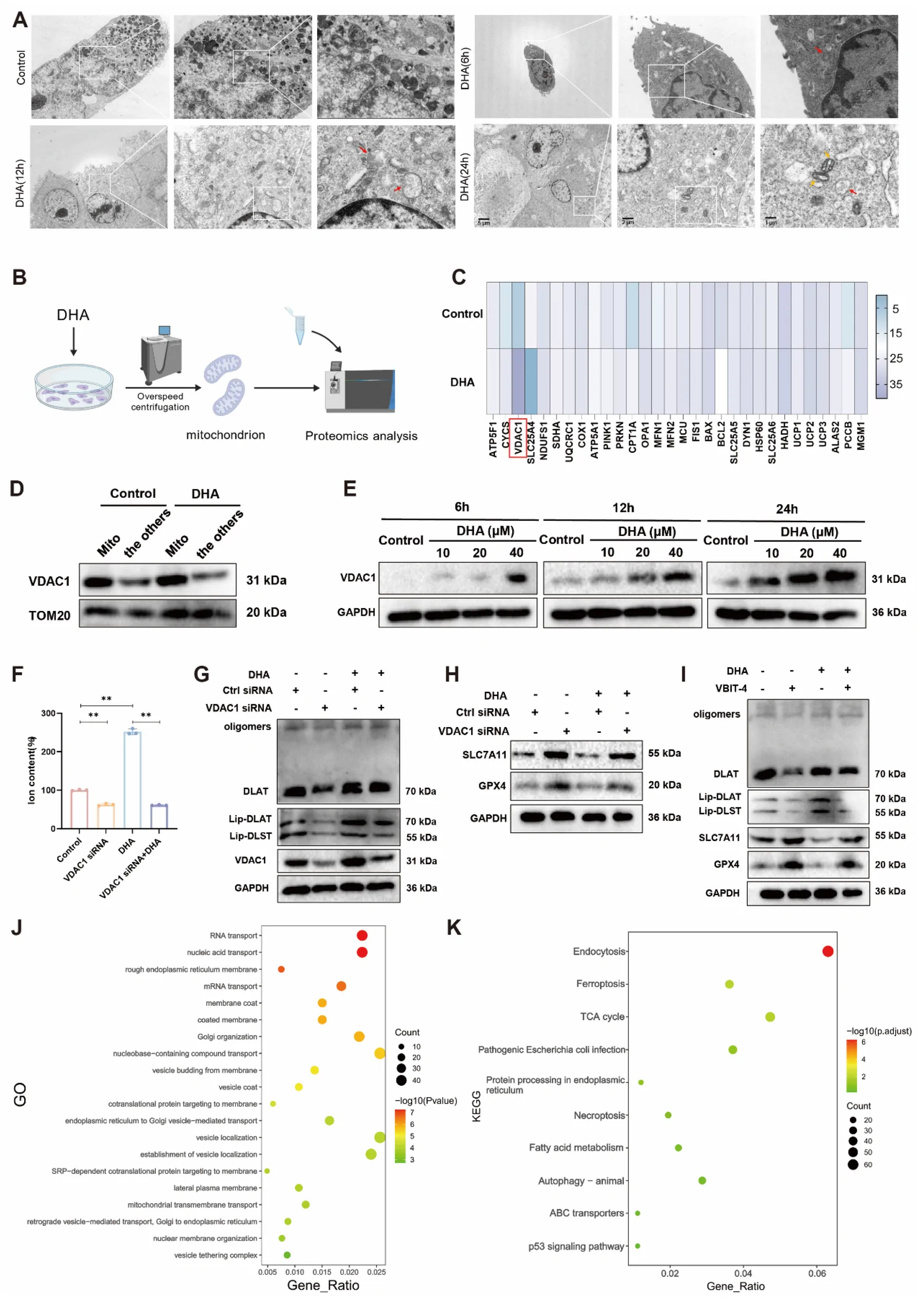
** DHA mediates mitochondrial copper import and death signal amplification through VDAC1.** (A) TEM images were taken after LX2 cells were treated with DHA (20 µM) for 6 h, 12 h, and 24 h. The image on the right shows an enlarged view of the boxed region in the left image. The red arrows indicate features associated with cuproptosis, while the yellow arrows highlight features related to ferroptosis. Scale bar: 5 µm. (B) After treatment with DHA (20 µM), mitochondria were extracted from LX2 cells for proteomic analysis. (C) Differential analysis of mitochondrial proteins after DHA (20 µM) treatment. (D) Western blot analysis was performed to examine the expression levels of VDAC1 in LX2 cells treated with DHA (20 µM) for 24 h, followed by gradient centrifugation. Protein expression levels were quantified using grayscale analysis (n = 3). (E) Western blot analysis was used to detect the expression levels of VDAC1 in LX2 cells treated with DHA (0-40 µM) for 6 h, 12 h, and 24 h. Protein levels were quantified using grayscale analysis (n = 3). (F) ICP-MS was performed to detect mitochondrial copper ion content in LX2 cells treated with DHA (20 μM) for 24 hours and transfected with either VDAC1 siRNA or negative control siRNA (n = 3). (G) Western blot analysis was performed to assess the expression levels of oligomerized DLAT, Lip-DLAT, Lip-DLST, and VDAC1 proteins in LX2 cells treated with DHA (20 µM) for 24 hours, followed by transfection with VDAC1 siRNA or negative control siRNA. Protein levels were quantified using grayscale analysis (n = 3). (H) Western blot analysis was used to detect the expression levels of SLC7A11 and GPX4 proteins in LX2 cells treated with DHA (20 µM) for 24 hours, followed by transfection with VDAC1 siRNA or negative control siRNA. Protein expression was quantified using grayscale analysis (n = 3). (I) Western blot was performed to detect the expression levels of oligomerized DLAT protein, Lip-DLAT, Lip-DLST, SLC7A11, and GPX4 in LX2 cells treated with the combination of VBIT-4 (5 μM) and DHA (20 μM) for 24 hours, with quantification by densitometric analysis (n = 3). (J-K) IP/MS was used to identify the protein interaction network associated with VDAC1 in LX2 cells. The data were further analyzed by GO and KEGG pathway analysis (n = 3). Data are presented as mean ± SD, with p-values calculated using one-way ANOVA. ns, not significant; *p < 0.05, **p < 0.01.

To validate the DHA-induced cell death process, transmission electron microscopy (TEM) was employed to observe ultrastructural changes in cells at 6 h, 12 h, and 24 h post-DHA intervention. Combining previous studies with our experimental results^42^, we observed significant mitochondrial swelling, dissociation of inner and outer membranes, complete disintegration of cristae structures, and massive accumulation of dense granules at all three time points, presenting typical ultrastructural characteristics of cuproptosis. By 24 h, in addition to the aforementioned cuproptosis features, typical ferroptosis morphological alterations were also evident, including marked mitochondrial shrinkage, reduced cristae number, and membrane condensation (Figure 3A). This chronological pattern further confirms that DHA treatment initially induces cuproptosis in HSCs, subsequently triggering ferroptosis as time progresses.

To identify the key mitochondrial regulatory molecules in DHA-induced cuproptosis and ferroptosis, mitochondrial fractions were isolated from LX2 cells by ultracentrifugation following 24 h of DHA intervention for proteomic analysis (Figure 3B). The results demonstrated that DHA treatment significantly altered the mitochondrial protein expression profile, with the mitochondrial transmembrane protein Voltage-Dependent Anion Channel 1 (VDAC1) showing the most pronounced difference (Figure 3C), suggesting its potential as a critical target in DHA-induced cuproptosis and ferroptosis. In this study, mitochondrial enrichment prior to proteomic analysis effectively avoided cytoplasmic signal interference inherent in whole-cell proteomics, ensuring accurate identification of mitochondria-specific alterations. Mitochondrial isolation by ultracentrifugation was further performed to validate the mitochondrial localization of VDAC1, using the outer mitochondrial membrane protein TOM20 as a reference. The results indicated that VDAC1 protein expression was significantly upregulated in mitochondria following DHA treatment (Figure 3D, Supplementary Fig. S3A). To investigate the temporal regulatory effects of DHA on VDAC1 expression, LX2 cells were treated with gradient concentrations of DHA for 6 h, 12 h, and 24 h, followed by detection of VDAC1 protein levels. Chronological analysis revealed sustained upregulation of VDAC1 from 6 h to 24 h, which correlated temporally with intracellular copper and iron accumulation (Figure 3E, Supplementary Fig. S3B-D), suggesting that VDAC1 may play a pivotal role in DHA-induced cuproptosis and ferroptosis by mediating metal ion metabolism.

Subsequently, to investigate the potential role of VDAC1 in DHA-induced cuproptosis and ferroptosis, we examined changes in relevant indicators through targeted interference of VDAC1 expression. ICP-MS analysis of mitochondrial copper content revealed that VDAC1 knockdown significantly attenuated DHA-induced mitochondrial copper accumulation (Figure 3F). Furthermore, interference of VDAC1 expression markedly suppressed DHA-induced cuproptosis and ferroptosis, as evidenced by significantly decreased expression of lipoic acid-modified proteins and DLAT, along with upregulated expression of SLC7A11 and GPX4. DHA treatment significantly upregulated lipoylated proteins and induced DLAT oligomerization, while concomitantly downregulating SLC7A11 and GPX4. In contrast, VDAC1 silencing markedly reversed these alterations (Figure 3G-H, Supplementary Fig. S3E-F). Cell viability assays using CCK-8 demonstrated that VDAC1 knockdown significantly reversed the decreased cell viability induced by DHA or Elesclomol-Cu (Supplementary Fig. S3G). We further validated VDAC1 function using the VDAC1 channel inhibitor VBIT-4. Experimental results showed that both VBIT-4 and VDAC1 siRNA significantly reversed DHA-induced cuproptosis and ferroptosis, indicating that the channel function of VDAC1 constitutes the primary mechanism mediating cuproptosis (Figure 3I, Supplementary Fig. S3H).

As VDAC1 is one of the main channels for material exchange between the mitochondria and the cytoplasm, it plays a crucial role in cellular energy metabolism, ion homeostasis regulation, and cell death signaling^43^, ^44^. To investigate the upstream regulatory mechanism by which DHA modulates VDAC1-mediated synergistic cuproptosis and ferroptosis, we employed co-immunoprecipitation coupled with mass spectrometry (IP/MS) to systematically identify the protein interaction network associated with VDAC1. The analysis revealed that VDAC1 primarily interacts with proteins involved in intracellular transport, vesicle trafficking, and Golgi apparatus-related functions. GO enrichment analysis demonstrated that these interacting proteins were significantly enriched in biological processes including membranous organelle transport, vesicle-mediated cargo transport, and regulation of mitochondrial outer membrane permeability (Figure 3J). KEGG pathway analysis further revealed their involvement in endocytosis, mitochondrial energy metabolism, apoptotic signaling, and metal ion metabolic regulation (Figure 3K). Reactome pathway analysis also elucidated their close association with intracellular transport and vesicle trafficking. These results collectively indicate that VDAC1 may regulate cellular material exchange and membrane structural stability through interactions with various transport-related proteins (Supplementary Fig. S3I).

In summary, VDAC1 may serve as a critical effector molecule in DHA-induced cuproptosis and ferroptosis in HSCs, and further amplifies this process through interactions with various transport-related proteins.

### 2.4 DHA Targets RAB1B to Activate Cuproptosis and Ferroptosis through Mechanotransduction-Mediated VDAC1 Opening

**Figure 4:**
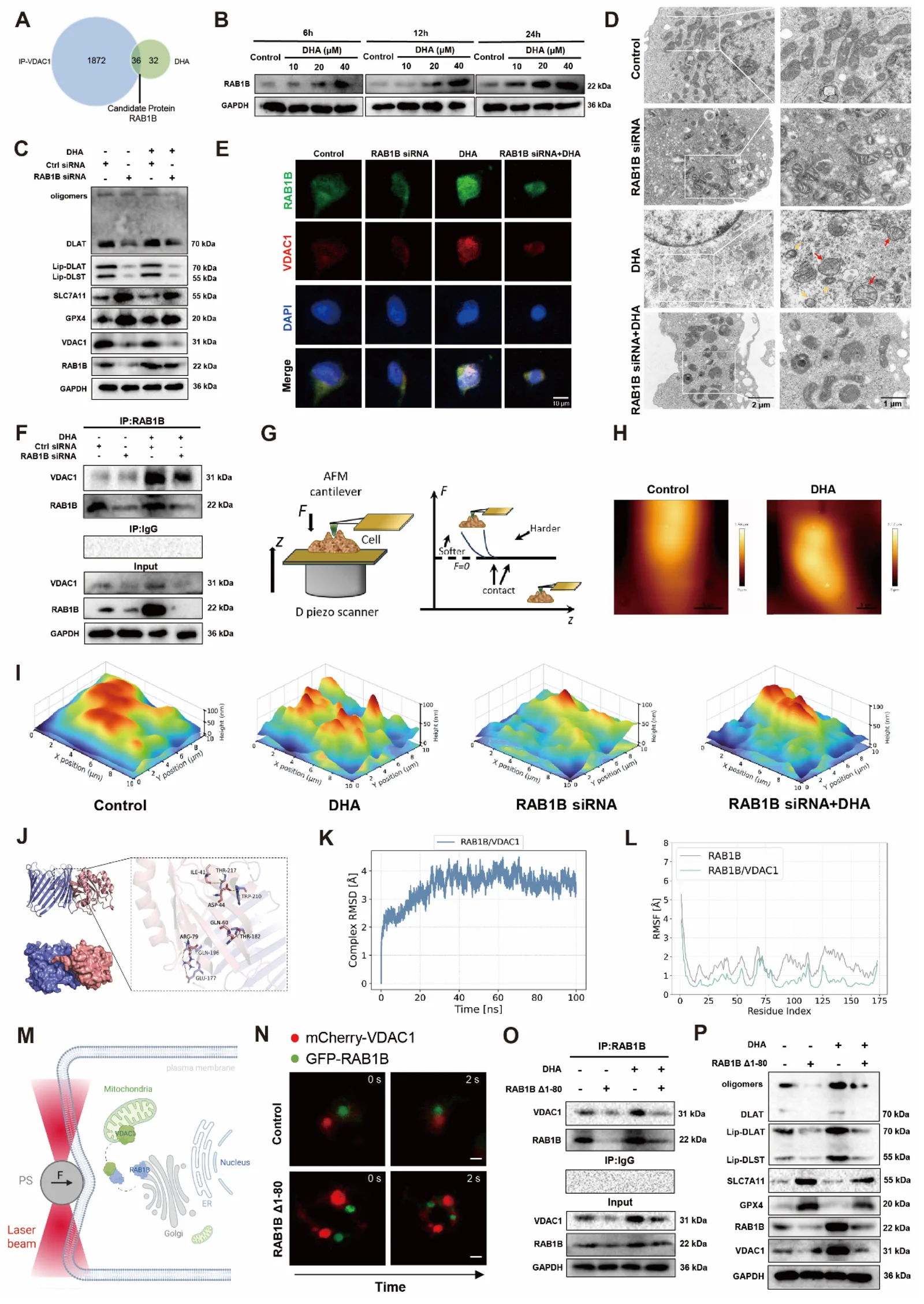
** DHA targets RAB1B to activate cuproptosis and ferroptosis through mechanotransduction-mediated VDAC1 opening.** (A) Differential protein intersection analysis. (B) Western blot analysis was performed to assess RAB1B protein expression following DHA (0-40 μM) treatment for 6 h, 12 h, and 24 h, with quantification via densitometric analysis (n=3). (C) Western blot analysis of oligomerized DLAT, Lip-DLAT, Lip-DLST, SLC7A11, GPX4, RAB1B, VDAC1 protein expression in LX2 cells treated with DHA (20 µM) for 24 h, followed by transfection with RAB1B siRNA or negative control siRNA. Protein expression was quantified using grayscale analysis (n = 3). (D) TEM was used to observe the microscopic features of copper and ferroptosis in LX2 cells treated with DHA (20 µM) for 24 h and transfected with RAB1B siRNA or negative control siRNA The red arrows indicate features associated with cuproptosis, while the yellow arrows highlight features related to ferroptosis. Scale bar: 2 µm. (E) Immunofluorescence was conducted to observe the co-localization of RAB1B and VDAC1 in LX2 cells treated with DHA (20 µM) and transfected with RAB1B siRNA or negative control siRNA (n = 3). (F) Co-IP was performed to detect the interaction between RAB1B and VDAC1 in LX2 cells treated with DHA (20 µM) and transfected with RAB1B siRNA or negative control siRNA (n = 3). (G) Schematic diagram illustrating AFM measurements. (H) AFM height topography images of LX2 cells treated with DHA (20 µM). Scale bar: 5 µm. (I) Representative 3D images of LX2 ultrastructural features. (J) Binding mode of the RAB1B-VDAC1 complex. Pink represents RAB1B and blue represents VDAC1 protein. (K) RMSD of the RAB1B-VDAC1 complex during molecular dynamics simulations over time. (L) RMSF calculated based on molecular dynamics simulation trajectories. (M) Schematic diagram illustrating the interaction between RAB1B and VDAC1 observed using optical tweezers combined with confocal microscopy. (N) Confocal fluorescence microscopy of the co-localization of mutated RAB1B N-terminal 1-80 aa domain with VDAC1. Scale bar: 10 µm. (O) Co-IP analysis of the RAB1B-VDAC1 interaction in LX2 cells transfected with the RAB1B N-terminal 1-80 amino acid deletion mutant and treated with DHA (20 µM) (n = 3). (P) Western blot analysis of oligomerized DLAT, Lip-DLAT, Lip-DLST, SLC7A11, GPX4, RAB1B, VDAC1 protein expression levels in LX2 cells treated with DHA (20 µM) and mutated RAB1B N-terminal 1-80 aa domain. Protein expression was quantified using grayscale analysis (n = 3). Data are presented as mean ± SD, with p-values calculated using one-way ANOVA. ns, not significant; *p < 0.05, **p < 0.01.

To further identify upstream regulators of VDAC1, an intersection analysis was performed between VDAC1-interacting proteins and differentially expressed proteins from the whole proteome of LX2 cells following DHA treatment. RAB1B was identified as the most likely key target capable of binding DHA and modulating VDAC1 function (Figure 4A, Supplementary Fig. S4A). Experimental results demonstrated that DHA dose-dependently upregulated RAB1B expression at all stages (Figure 4B, Supplementary Fig. S4B). We simultaneously detected the basal expression abundance of RAB1B in primary hepatocytes and primary HSCs, and the results verified that the basal expression of RAB1B was markedly higher in HSCs than in hepatocytes. After DHA treatment, the upregulation magnitude of RAB1B in HSCs was significantly stronger than that in hepatocytes. Collectively, HSCs exhibited both higher basal RAB1B expression and a more prominent drug-induced upregulation response (Supplementary Fig. S2A-B). Further analysis revealed that RAB1B knockdown significantly suppressed DHA-induced cuproptosis and ferroptosis processes, while concurrently downregulating VDAC1 protein levels (Figure 4C, Supplementary Fig. S4C-D). Meanwhile, RAB1B knockdown significantly reversed the decreased cell viability induced by DHA or Elesclomol-Cu (Supplementary Fig. S4E). TEM observations showed that RAB1B knockdown rescued the ultrastructural alterations of cuproptosis and ferroptosis induced by DHA (Figure 4D). Immunofluorescence staining was performed to examine the expression and subcellular localization of RAB1B and VDAC1. Results showed that RAB1B silencing significantly attenuated DHA-induced VDAC1 upregulation and altered its subcellular localization (Figure 4E, Supplementary Fig. S4F). Subsequently, Co-IP experiments confirmed the existence of an interaction between RAB1B and VDAC1 (Figure 4F). These findings collectively suggest that DHA may promote copper and iron ion accumulation and induce cell death by upregulating RAB1B expression and enhancing its colocalization with VDAC1.

Cuproptosis and ferroptosis are often associated with membrane rupture and cellular structural collapse, which can lead to significant changes in cell morphology and mechanical properties^13^. To investigate the effects of DHA on the physical properties of HSC membranes, we performed force spectroscopy analysis on cells using atomic force microscopy (AFM) (Figure 4G). DHA treatment altered HSC morphology and mechanical properties. Compared with the control group, cell height increased from 1.56 μm to 2.17 μm, whereas Young's modulus decreased from 1.46 ± 0.17 kPa to 0.51 ± 0.10 kPa. These findings indicate that DHA significantly reduces cell stiffness and softens the plasma membrane (Figure 4H, Supplementary Fig. S4G-H). RAB1B knockdown restored Young's modulus to 2.20 ± 0.12 kPa, thereby reversing the softening effect of DHA (Supplementary Fig. S4G-H). This demonstrates that RAB1B serves as a key mediator of DHA-induced modulation of HSC mechanical properties. Further analysis revealed that DHA treatment markedly increased nanoscale surface roughness, and this scale approximated the dimensions of plasma membrane caveolae. Concomitantly, RAB1B knockdown reversed these membrane dynamics (Figure 4I). Based on these observations, we hypothesize that DHA modulates cellular mechanical properties through membrane remodeling processes associated with vesicular trafficking.

Based on the membrane mechanical changes observed by AFM, we hypothesized that RAB1B may regulate VDAC1 function by sensing or responding to alterations in the membrane environment. We analyzed the interaction mechanism between RAB1B and VDAC1 using molecular docking and molecular dynamics simulation techniques. Molecular docking results revealed that RAB1B primarily binds to VDAC1 through its N-terminal 1-80 amino acid sequence (Figure 4J). Subsequently, we performed structural stability and energetic analyses of the RAB1B-VDAC1 complex using MD simulations. During the 100 ns MD simulation, a stable binding interface formed between RAB1B and VDAC1, with RMSD values stabilizing after approximately 30 ns, indicating that the complex maintains a stable conformation over extended timescales (Figure 4K). RMSF analysis demonstrated that the flexibility of the RAB1B N-terminal region was significantly reduced upon binding to VDAC1, while key binding residues (such as ILE41, ASP44, GLN60, ARG79, etc.) formed multiple stable hydrogen bonds and salt bridge interactions with THR182, TRP210, and GLU177 of VDAC1, thereby enhancing the structural stability of the complex (Figure 4L). Additionally, changes in the radius of gyration (RoG) also supported this conclusion (Supplementary Fig. S4I). Regarding binding energy analysis, the MM/GBSA method calculated a binding free energy of -50.11 ± 3.45 kcal/mol, indicating strong binding affinity between the two proteins (Supplementary Fig. S4J). Meanwhile, the number of hydrogen bonds in the RAB1B-VDAC1 complex remained stable between 3-7, with occasional transient decreases that rapidly recovered to the stable range (Supplementary Fig. S4K). Molecular dynamics also revealed the top 10 residues contributing most significantly to the binding energy of their interaction (Supplementary Fig. S4L). These results collectively suggest that RAB1B and VDAC1 possess a stable and specific binding mode that may be regulated by the mechanical environment.

To further validate these findings at the cellular level, we used optical tweezers combined with confocal microscopy to assess the mechanical response of the RAB1B-VDAC1 interaction. We tagged RAB1B and VDAC1 with GFP-RAB1B (green) and mCherry-VDAC1 (red), respectively, and applied small compressive forces of 0.1-10 nN to specific regions of the cell membrane using optical tweezers. The localization changes of RAB1B and VDAC1 under mechanical stimulation were monitored in real time by confocal microscopy (Figure 4M). The results showed that, under normal conditions, mechanical stimulation significantly enhanced the co-localization signal of RAB1B and VDAC1, leading to their spatial aggregation, indicating that external force facilitates their binding and activation. However, when the key binding domain of RAB1B was mutated, the aggregation signal of VDAC1 was significantly reduced, and co-localization disappeared, suggesting that the integrity of RAB1B's key structural domain is crucial for the mechanical activation of VDAC1 (Figure 4N). Co-IP assays further demonstrated that deletion of amino acids 1-80 at the N-terminus of RAB1B markedly impaired this interaction (Figure 4O). To further verify the functional consequences of RAB1B-mediated mechanical regulation, we assessed the expression changes of key proteins after DHA intervention by Western blot. The results showed that the deletion of the N-terminal 1-80 amino acid segment of RAB1B significantly reversed DHA-induced cuproptosis and ferroptosis, while also significantly reducing the expression of VDAC1 (Figure 4P, Supplementary Fig. S4M).

In summary, RAB1B senses and transduces mechanical signals through its N-terminal domain, enhancing the interaction between RAB1B and VDAC1, thereby leading to sustained amplification of cuproptosis and ferroptosis signaling.

### 2.5 DHA Promotes Vesicular Endocytosis-Driven Copper Ion Transport through RAB1B under Copper Overload

**Figure 5:**
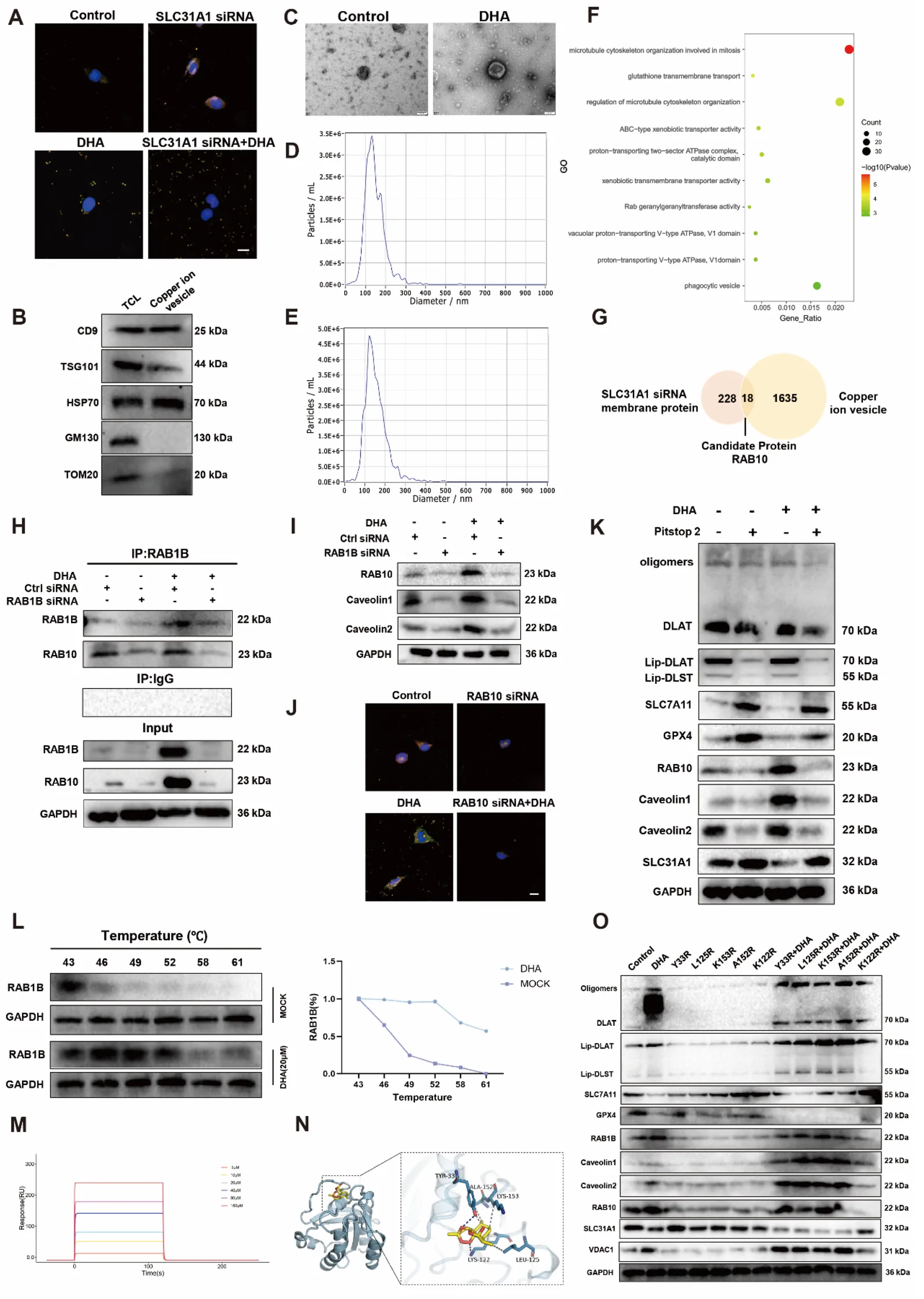
** DHA promotes vesicular endocytosis-driven copper ion transport through RAB1B under copper overload.** (A) Immunofluorescence analysis of vesicle expression levels in LX2 cells treated with DHA (20 µM) for 24 h and transfected with SLC31A1 siRNA or negative control siRNA (n = 3). Scale bar: 20 µm. (B) Western blot analysis of vesicular markers (CD9, TSG101, and HSP70), Golgi marker GM130, and mitochondrial marker TOM20 in copper ion vesicles and total cell lysate. (C) TEM images of copper ion vesicles. (D-E) NTA of the particle size distribution of copper ion vesicles in the control group and the DHA (20 µM) treatment group. (F) IP/MS identification of the protein interaction network associated with copper ion vesicles, followed by GO analysis (n = 3). (G) Protein interaction network analysis of membrane proteins and copper ion vesicles in LX2 cells transfected with SLC31A1 siRNA, based on proteomics differential protein intersection analysis. (H) Co-IP analysis of the interaction between RAB10 and RAB1B in LX2 cells treated with DHA (20 µM) for 24 h and transfected with RAB1B siRNA or negative control siRNA (n = 3). (I) Western blot analysis of RAB10, Caveolin1, Caveolin2 protein expression levels in LX2 cells treated with DHA (20 µM) for 24 h and transfected with RAB1B siRNA or negative control siRNA. Expression levels were quantified using grayscale analysis (n = 3). (J) Immunofluorescence analysis of vesicle expression levels in LX2 cells treated with DHA (20 µM) for 24 hours and transfected with RAB10 siRNA or negative control siRNA (n = 3). Scale bar: 20 µm. (K) Western blot analysis of the expression levels of GPX4, SLC7A11, RAB10, Caveolin1, Caveolin2, Lip-DLST, Lip-DLAT, and oligomerized DLAT proteins in LX2 cells treated with DHA (20 µM) for 24 hours, with or without Pitstop-2 (20 µM) treatment. Expression levels were quantified using grayscale analysis (n = 3). (L) The binding affinity between DHA and RAB1B protein in LX2 cells treated with DHA (20 μM) for 24 hours was detected by cellular thermal shift assay (n=3). (M) SPR was used to evaluate the binding affinity between DHA and RAB1B. (N) Molecular docking analysis of potential binding sites between RAB1B protein and DHA. (O) Western blot analysis of protein expression levels of RAB1B, SLC31A1, VDAC1, GPX4, SLC7A11, RAB10, Caveolin1, Caveolin2, Lip-DLST, Lip-DLAT, and oligomerized DLAT in LX2 cells transfected with RAB1B binding site mutant constructs followed by DHA (20 µM) treatment for 24 h. Expression levels were quantified using grayscale analysis (n = 3). Data are presented as mean ± SD, with p-values calculated using one-way ANOVA. ns, not significant; *p < 0.05, **p < 0.01.

Based on previous findings, DHA treatment induced cuproptosis accompanied by intracellular copper overload. The plasma membrane exhibited markedly enhanced nanoscale surface roughness, with dimensions approximating those of typical caveolar invaginations. Concomitantly, the elastic modulus decreased and mechanical properties were altered. Caveolae, as flask-shaped microdomains formed by plasma membrane invagination, are highly sensitive to membrane tension changes during their formation and internalization; membrane softening promotes the transition from static conformations to dynamic invaginations, thereby driving vesicle formation. Furthermore, examination of caveolin protein expression revealed no significant changes at 0-12 h following DHA treatment, whereas dose-dependent upregulation was observed at 24 h. These findings indicate that the caveolin-mediated endocytic pathway is progressively activated under sustained copper overload (Supplementary Fig. S5A). Based on these observations, it is hypothesized that intracellular copper overload may trigger activation of the caveolin-dependent endocytic pathway as an alternative copper uptake mechanism to compensate for SLC31A1-mediated copper transport. To validate this hypothesis, SLC31A1 was silenced and cells were subsequently treated with DHA. Results showed that SLC31A1 knockdown significantly promoted vesicle formation (Figure 5A). ICP-MS analysis further revealed that DHA treatment alone elevated intracellular copper levels; notably, combined SLC31A1 knockdown and DHA treatment resulted in a significant increase in copper content compared with DHA treatment alone (Supplementary Fig. S5B). These findings confirm that the endocytic pathway mediates copper ion uptake in a compensatory manner.

To characterize the molecular composition and functional features of DHA-induced endocytic vesicles, we isolated endocytic vesicles from LX2 cells by ultracentrifugation, followed by specific dye labeling and flow cytometry sorting to enrich copper-containing vesicles. Western blot analysis was performed on the isolated vesicles. The results showed that the vesicles were positively stained for CD9, TSG101, and HSP70, three established vesicle marker proteins, while the negative marker proteins GM130 and TOM20 were undetectable (Figure 5B). These copper-enriched vesicles were characterized by TEM (Figure 5C). Nanoparticle Tracking Analysis (NTA) results showed that vesicle diameters in both control and DHA-treated groups were predominantly distributed between 100-200 nm, consistent with the physical characteristics of typical endocytic vesicles. However, compared to the control group, the DHA-treated group exhibited significantly increased particle number density and markedly elevated peak concentration, indicating enhanced vesicle generation and secretory activity following DHA stimulation. Further analysis revealed that the DHA group showed a slight shift in the peak diameter distribution toward smaller sizes, suggesting that DHA may induce force-mediated membrane reorganization and promote higher-frequency endocytosis and vesicle budding events (Figure 5D-E). Subsequently, proteomic analysis was performed on the isolated copper-enriched vesicles. GO enrichment analysis demonstrated that these differentially expressed proteins were significantly enriched in biological processes closely associated with cytoskeletal organization, RAB family-mediated vesicular transport, and ion transport (Figure 5F). Reactome pathway analysis further revealed significant enrichment of differential proteins in multiple pathways related to intracellular cargo transport and processing (Supplementary Fig. S5C).

Simultaneously, we performed intersection analysis between the proteomic data of copper-enriched vesicles and the membrane proteomic data extracted after SLC31A1 interference to screen for key molecules commonly enriched in both datasets. RAB10, which was significantly enriched in both proteomic datasets, was identified as a potential core regulatory molecule in copper ion transport and endocytic processes (Figure 5G). RAB10, a small GTPase in the RAB family, is widely involved in vesicular trafficking, cell membrane remodeling, and the regulation of endocytic pathways^45^. RAB10 typically works in conjunction with other RAB proteins to mutually regulate their activity and membrane binding, coordinating multiple cellular processes^46^. Based on this, we further validated the molecular interaction network of RAB10 through Co-IP. The results demonstrated a stable interaction between RAB10 and RAB1B (Figure 5H). Subsequently, we found that RAB1B knockdown significantly downregulated the expression of vesicular endocytosis-related regulatory proteins, whereas DHA treatment reversed this phenomenon (Figure 5I, Supplementary Fig. S5D). Building upon these findings, we further investigated the regulatory role of RAB10 in endocytic vesicles. Immunofluorescence results showed that RAB10 knockdown significantly inhibited DHA-induced vesicle formation (Figure 5J). Additionally, we treated cells with the combination of endocytosis inhibitor Pitstop-2 and DHA. The results indicated that Pitstop-2 treatment suppressed both cuproptosis and ferroptosis processes, accompanied by downregulation of endocytosis-related protein expression; however, DHA treatment reversed these effects and restored the expression levels of relevant proteins (Figure 5K, Supplementary Fig. S5E).

RAB1B plays a pivotal role in the synergistic mechanism of DHA-mediated cuproptosis and ferroptosis. We further assessed the binding affinity between DHA and RAB1B using the CETSA. The results showed that the thermal stability of RAB1B was significantly enhanced in the DHA-treated group, indicating that DHA can directly bind to RAB1B and increase its thermal stability (Figure 5L). SPR further demonstrated favorable binding between DHA and RAB1B (Figure 5M). To gain deeper insights into the specific binding mechanism between RAB1B and DHA, we performed molecular docking to analyze the binding sites of RAB1B protein with DHA. The molecular docking results revealed five potential binding sites between RAB1B and DHA: TYR33, ALA152, LYS153, LYS122, and LEU125 (Figure 5N). To investigate the specific roles of these binding sites in RAB1B-regulated cuproptosis and ferroptosis, we conducted site-directed mutagenesis of these five residues. Functional analysis of the mutant proteins showed that mutations at TYR33, ALA152, LYS153, and LEU125 did not significantly affect DHA-induced cuproptosis and ferroptosis, suggesting that these sites play limited roles in the interaction between RAB1B and DHA. Notably, mutation of LYS122 significantly reversed DHA-induced cuproptosis and ferroptosis effects, indicating that LYS122 plays a critical role in DHA binding to RAB1B and its biological effects (Figure 5O, Supplementary Fig. S5F).

In summary, DHA activates the vesicular endocytic pathway through the RAB1B-RAB10 axis, maintaining sustained copper ion uptake following copper overload-induced SLC31A1 degradation, thereby driving mitochondrial copper overload and subsequent cell death programs.

### 2.6 DHA Induces USP11 Succinylation to Promote SLC31A1 Ubiquitination and Degradation thereby Inhibiting SLC31A1-Dependent Copper Transport

**Figure 6:**
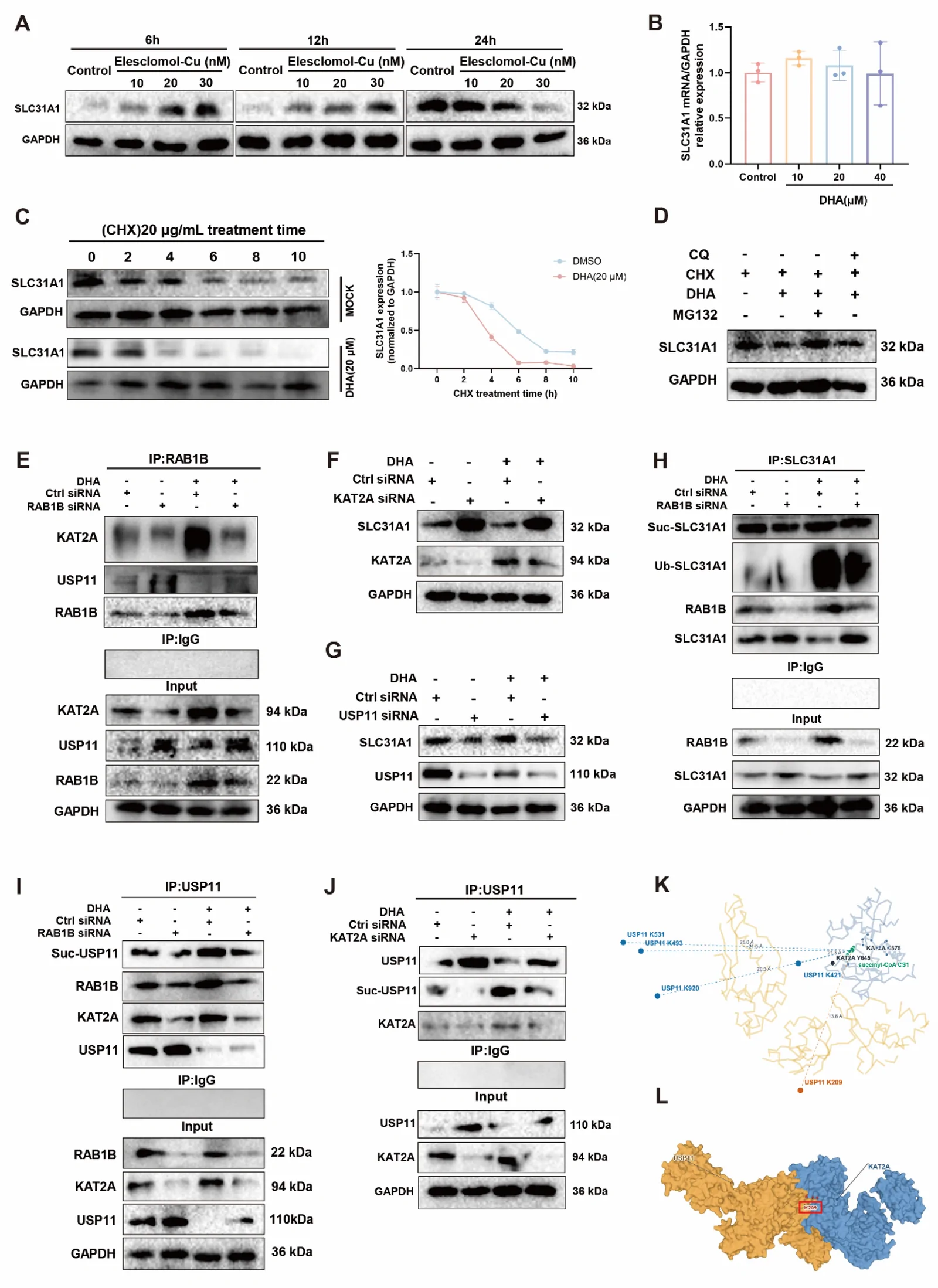
** DHA induces USP11 succinylation to promote SLC31A1 ubiquitination and degradation thereby inhibiting SLC31A1-dependent copper transport.** (A) Western blot analysis was conducted to assess the expression levels of SLC31A1 in LX2 cells treated with Elesclomol-Cu (0-30 nM) for 6 h, 12 h, and 24 h. Densitometric analysis was used for quantitative evaluation (n=3). (B) RT-qPCR analysis of SLC31A1 gene expression in LX2 cells treated with DHA (0-40 µM) for 24 h (n = 3). (C) Western blot analysis of the half-life of SLC31A1 protein in LX2 cells treated with DHA (20 µM) for 24 h, with or without protein synthesis inhibitors. Results were quantified using grayscale analysis (n = 3). (D) Western blot analysis of SLC31A1 protein expression in LX2 cells treated with DHA (20 µM) for 24 h, followed by treatment with CHX (20 µg/mL), MG132 (10 µM), CQ (5 µM), either alone or in combination. Protein expression was quantified using grayscale analysis (n = 3). (E) Co-IP analysis of interactions between RAB1B and KAT2A, USP11 in LX2 cells treated with DHA (20 µM) for 24 h and transfected with RAB1B siRNA or negative control siRNA (n = 3). (F) Western blot analysis of SLC31A1 protein expression in LX2 cells treated with DHA (20 µM) for 24 h, followed by transfection with KAT2A siRNA or negative control siRNA. Protein expression was quantified using grayscale analysis (n = 3). (G) Western blot analysis of SLC31A1 protein expression in LX2 cells treated with DHA (20 µM) for 24 h, followed by transfection with USP11 siRNA or negative control siRNA. Protein expression was quantified using grayscale analysis (n = 3). (H) Immunoprecipitation of SLC31A1 followed by Western blot analysis of ubiquitination and succinylation levels in LX2 cells treated with DHA (20 µM) for 24 h (n = 3). (I) Co-IP analysis of interactions between RAB1B and USP11 in LX2 cells transfected with RAB1B siRNA or negative control siRNA, followed by DHA (20 µM) treatment for 24 h, and assessment of USP11 succinylation levels (n = 3). (J) Co-IP analysis of interactions between KAT2A and USP11 in LX2 cells transfected with KAT2A siRNA or negative control siRNA, followed by DHA (20 µM) treatment for 24 h, and assessment of USP11 succinylation levels (n = 3). (K) Prediction of USP11 succinylation sites by protein-protein docking. (L) Surface model of protein-protein docking between KAT2A (blue) and USP11 (orange). Data are presented as mean ± SD, with p-values calculated using one-way ANOVA. ns, not significant; *p < 0.05, **p < 0.01.

To investigate the temporal changes in copper ion transport-related proteins, we treated LX2 cells with Elesclomol-Cu complexes as a copper ion donor at various time points and concentrations (0-30 nM). The results revealed a significant increase in SLC31A1 expression at 6 h and 12 h with increasing copper ion concentrations, followed by a notable decrease at 24 h (Figure 6A, Supplementary Fig. S6A). Consistently, this trend mirrored DHA-induced alterations in SLC31A1. These findings suggest that during the early stages of copper ion accumulation, cells upregulate SLC31A1 to facilitate copper uptake. However, as treatment duration and copper load increase, SLC31A1 undergoes a self-limiting downregulation. Previous observations revealed that expression of the copper efflux-related protein ATP7B exhibited no significant time-dependent alterations, indicating that during the dynamic changes in copper load induced by DHA, LX2 cells primarily regulate copper homeostasis by modulating SLC31A1-mediated copper uptake, rather than relying on the ATP7B-mediated efflux pathway.

To further elucidate the degradation mechanism of SLC31A1 under DHA intervention, gene expression analysis confirmed that DHA treatment did not significantly affect the mRNA levels of SLC31A1, suggesting that the changes in its expression may be due to alterations in protein stability rather than transcriptional regulation (Figure 6B). Consequently, we investigated the impact of DHA on SLC31A1 protein stability. After treating the cells with the protein synthesis inhibitor cycloheximide (CHX), we found that DHA significantly shortened the half-life of SLC31A1 protein in LX2 cells, indicating that DHA accelerates the degradation of SLC31A1 (Figure 6C). Considering that the autophagy-lysosomal pathway and the ubiquitin-proteasome system are two major mechanisms of protein degradation, we further explored whether DHA reduces SLC31A1 expression by promoting its ubiquitination or autophagy. The results showed that MG-132, a 26S proteasome inhibitor, significantly blocked DHA-induced downregulation of SLC31A1 protein, while CQ treatment did not restore its expression (Figure 6D, Supplementary Fig. S6B). These findings suggest that DHA reduces SLC31A1 protein levels via a ubiquitination-dependent proteolytic pathway rather than through the autophagy-lysosomal system.

Building on previous findings that RAB1B is dose-dependently upregulated over time, we hypothesize that RAB1B may mediate the observed changes in SLC31A1 expression. To investigate how RAB1B regulates the degradation of SLC31A1, we interfered with RAB1B expression following DHA treatment and assessed SLC31A1 protein levels. The results showed that DHA treatment significantly reduced SLC31A1 expression, and this effect was reversed upon RAB1B interference (Supplementary Fig. S6C). Additionally, our proteomic analysis following DHA treatment revealed an increase in SUCLA2 expression (Supplementary Fig. S4A). SUCLA2, a subunit of succinyl-CoA synthetase, is upregulated, which may indicate alterations in the cellular metabolic or modification state. To further identify potential regulatory factors involved in RAB1B-mediated SLC31A1 homeostasis, we performed CO-IP/MS to analyze RAB1B interactions. We then conducted intersection analysis between the identified interacting proteins and the differentially expressed proteins from global proteomics of DHA-treated LX2 cells. The results revealed that the deubiquitinase USP11 and the lysine succinyltransferase KAT2A interact with RAB1B, suggesting that both enzymes may serve as key regulators within the RAB1B signaling pathway, modulating the expression and stability of SLC31A1 (Supplementary Fig. S6D).

Co-IP assays further demonstrated interactions between RAB1B and USP11, as well as KAT2A (Figure 6E). We next interfered with the expression of KAT2A and USP11 to assess their impact on SLC31A1 homeostasis. The results showed that KAT2A silencing significantly upregulated SLC31A1 protein levels, whereas DHA treatment markedly reversed this effect (Figure 6F, Supplementary Fig. S6E). In contrast, interference with USP11 expression resulted in a significant decrease in SLC31A1 protein levels (Figure 6G, Supplementary Fig. S6F). These findings indicate that KAT2A and USP11 exert antagonistic effects in the regulation of SLC31A1. We then examined the post-translational modification status of SLC31A1 after 24 h of DHA treatment. The results showed that DHA significantly enhanced the ubiquitination of SLC31A1, while SLC31A1's succinylation levels remained unchanged (Figure 6H). This suggests that DHA-induced SLC31A1 degradation primarily relies on the ubiquitination pathway. Given that SLC31A1's succinylation was unaffected, we hypothesize that DHA may influence USP11 activity by altering its modification status. Further investigation confirmed that DHA induces an increase in USP11 succinylation, accompanied by a decrease in its protein expression (Figure 6I). In contrast, interference with KAT2A significantly inhibited USP11 succinylation and restored its protein levels (Figure 6J), which identifies KAT2A as a key enzyme mediating USP11 succinylation. To precisely map modification sites, protein-protein docking coupled with succinyl-CoA pocket projection analysis was employed to assess the spatial accessibility of USP11 candidate lysine residues relative to the KAT2A catalytic center. Results showed that USP11 K209 exhibited significantly higher spatial accessibility than other candidate sites (Figure 6K-L). Furthermore, mass spectrometry analysis revealed that Lys209 (K209) served as the primary succinylation site (Supplementary Fig. S6G-H). Finally, functional validation experiments demonstrated that USP11 silencing enhanced SLC31A1 ubiquitination levels (Supplementary Fig. S6I).

In conclusion, DHA promotes the functional coupling of RAB1B with KAT2A and USP11, enhancing KAT2A-mediated USP11 succinylation, inhibiting USP11's deubiquitination activity, and accelerating SLC31A1 ubiquitination-dependent degradation.

### 2.7 Preparation and Characterization of DHA@HSCM-Lipo

**Figure 7:**
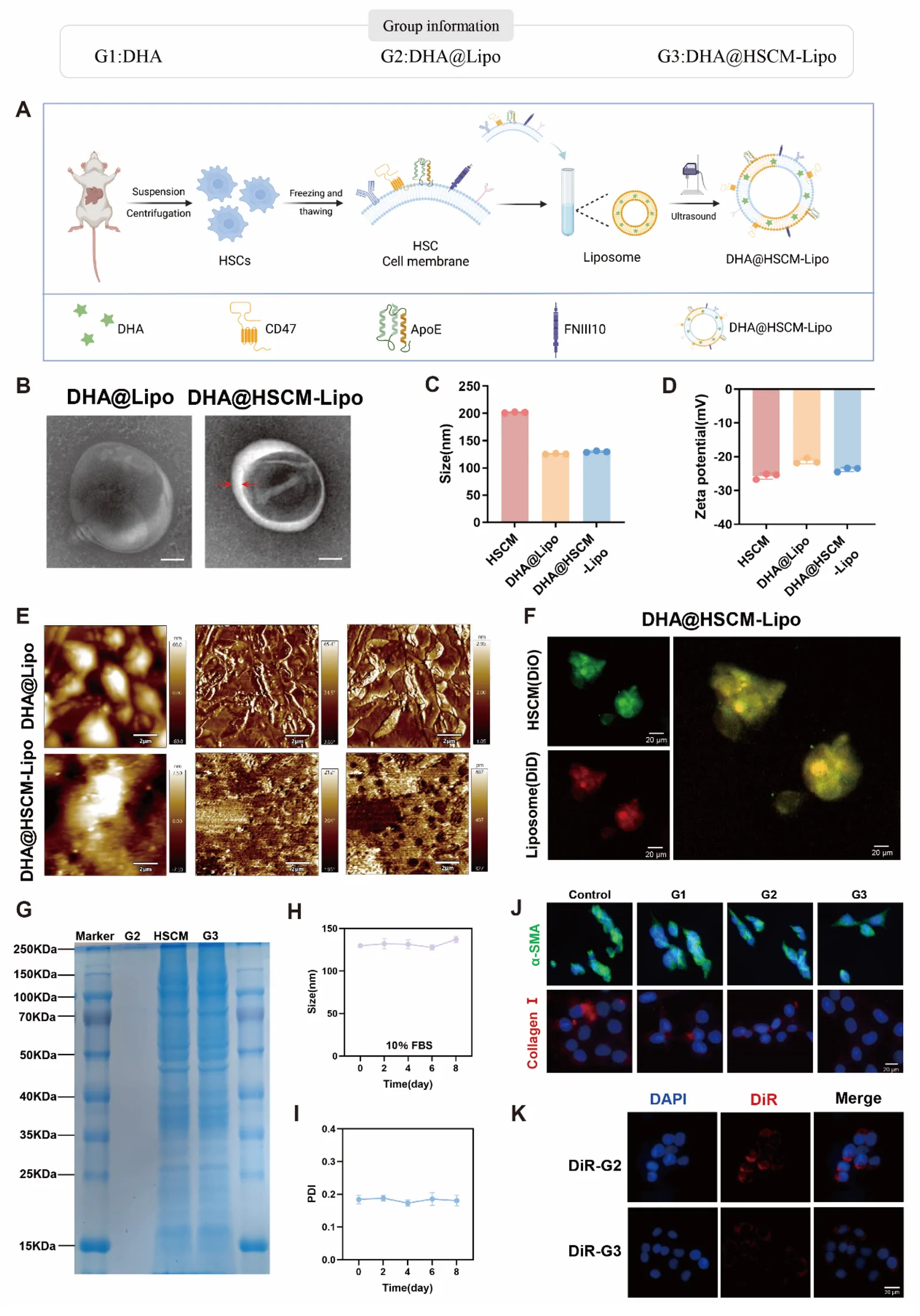
** Preparation and characterization of DHA@HSCM-Lipo.** (A) Schematic representation of the preparation process for DHA@HSCM-Lipo. (B) TEM images of DHA@Lipo and DHA@HSCM-Lipo. Scale bar: 100 nm. (C) Hydrodynamic size distribution of DHA@Lipo and DHA@HSCM-Lipo (n=3). (D) Zeta potential analysis of DHA@Lipo and DHA@HSCM-Lipo (n=3). (E) AFM images of DHA@Lipo and DHA@HSCM-Lipo. Scale bar: 2 µm. (F) Representative immunofluorescence confocal images showing co-localization of HSC membrane and liposomes. HSC membrane was labeled with DIO (green), and liposomes were labeled with DID (red). Scale bar: 20 µm. (G) SDS-PAGE analysis of total membrane proteins: HSC-Mem, DHA@Lipo, and DHA@HSCM-Lipo. (H) Particle size changes over time (0-8 days) in medium containing 10% FBS (n=3). (I) Particle PDI changes over time (0-8 days) in medium containing 10% FBS (n=3). (J) Immunofluorescence analysis of α-SMA (green) and Collagen I (red) expression in LX2 cells treated with DHA, DHA@Lipo, or DHA@HSCM-Lipo for 24 h, with cell nuclei stained with DAPI (blue). Scale bar: 20 µm. (K) Immunofluorescence detection of liposome uptake in RAW264.7 macrophages. Scale bar: 20 µm. Data are presented as mean ± SD, with p-values calculated using one-way ANOVA. ns, not significant; *p < 0.05, **p < 0.01.

The preparation process of the biomimetic liposome vesicles is illustrated in Figure 7A. First, DHA@Lipo was prepared using the thin-film hydration method. Subsequently, HSC membranes were extracted by ultracentrifugation, allowing them to fully integrate with the surface of DHA@Lipo, resulting in the construction of biomimetic liposomes coated with HSC membranes (DHA@HSCM-Lipo). The appearance of the liposome solution was shown in Supplementary Fig. S7A. For comparison, we also prepared DHA@Lipo without HSC membrane coating. Both types of liposomes were characterized and analyzed. TEM results revealed that both liposome formulations exhibited uniform spherical morphology (Figure 7B). DHA@HSCM-Lipo displayed more complex surface features, with a distinct core-shell structure, which may be related to the fusion of the cell membrane. Dynamic light scattering (DLS) analysis showed that the particle sizes of DHA@Lipo and DHA@HSCM-Lipo were approximately 100 nm and 120 nm, respectively (Figure 7C). The slightly larger particle size of DHA@HSCM-Lipo may be attributed to the cell membrane coating, which formed a more stable nanostructure following membrane fusion. Additionally, the zeta potentials of DHA@Lipo and DHA@HSCM-Lipo were -22.4 mV and -25.6 mV, respectively, indicating good stability of both liposomes under physiological conditions (Figure 7D). Further analysis of the surface morphology and uniformity of both liposomes was performed using AFM. AFM images revealed that, compared to DHA@Lipo, DHA@HSCM-Lipo exhibited a more uniform and smoother surface structure, with significantly lower surface roughness (Figure 7E). This suggests that the biomimetic cell membrane coating formed a more stable and uniform protective layer on the liposome surface, which may enhance liposome stability, cellular uptake efficiency, and biocompatibility *in vivo*. The entrapment efficiency (EE) and drug loading capacity (LC) of DHA in DHA@Lipo and DHA@HSCM-Lipo were assessed by high-performance liquid chromatography (HPLC). The entrapment efficiency of DHA in DHA@Lipo and DHA@HSCM-Lipo was 61.23% and 72.24%, respectively (Supplementary Fig. S7B), while the drug loading capacities were 10.93% and 15.66%, respectively (Supplementary Fig. S7C).

To further verify the fusion between HSC membranes and liposomes, we employed dual fluorescence labeling. DiO (green) was used to label the HSC membrane, while DiD (red) was used to label the liposomes. Confocal microscopy results showed excellent co-localization of the fluorescence signals, confirming the effective fusion between the HSC membrane and the liposomes (Figure 7F, Supplementary Fig. S7D). To assess the retention of membrane proteins during the preparation of DHA@HSCM-Lipo, we performed sodium dodecyl sulfate-polyacrylamide gel electrophoresis (SDS-PAGE) and Coomassie Brilliant Blue staining. The results showed that the protein band distribution in DHA@HSCM-Lipo was consistent with that of the HSC membrane, while no corresponding protein bands were detected in the uncoated DHA@Lipo, indicating successful modification of the liposomes with the HSC membrane and effective retention of membrane proteins (Figure 7G). Additionally, UV-visible spectroscopy indicated that the basic optical and molecular structural characteristics of DHA were largely retained (Supplementary Fig. S7E). Fourier transform infrared (FTIR) spectroscopy revealed characteristic absorption peaks related to membrane lipids, further verifying the successful encapsulation of the HSC membrane on the liposome surface and the maintenance of membrane integrity (Supplementary Fig. S7F).

To evaluate the stability of the liposomes, we stored them in culture medium containing 10% FBS for varying periods and assessed their stability at different time points. The results showed no significant changes in particle size and polydispersity index (PDI) from days 0 to 8 (Figures 7H-I), indicating that the liposomes exhibited good stability, making them suitable for long-term storage and experimental use. Further functional validation demonstrated that DHA@HSCM-Lipo significantly increased the protein expression of RAB1B, Caveolin-1, and Caveolin-2 (Supplementary Fig. S7G). To assess the functional effects of DHA@HSCM-Lipo in inhibiting HSC activation, we examined the expression of fibrosis marker proteins. Western blot results showed that DHA@HSCM-Lipo significantly reduced the protein levels of α-SMA and Collagen I in activated HSCs, with its inhibitory effect being significantly superior to that of DHA@Lipo or DHA alone (Supplementary Fig. S7H). Similar results were obtained from immunofluorescence experiments (Figure 7J). Nanoparticles were co-incubated with RAW264.7 macrophages. The cellular uptake rate in the DiR-G3 group was significantly lower than that in the DiR-G2 group. These findings indicate that DHA@HSCM-Lipo significantly reduces RAW264.7 macrophage uptake through membrane camouflage, thereby effectively evading phagocytic clearance (Figure 7K).

The successful construction of DHA@HSCM-Lipo not only preserves the biological characteristics of the cell membrane but also significantly enhances the stability and targeting ability of the nanodrug formulation. This provides an experimental foundation for the efficient delivery of DHA in the treatment of liver fibrosis.

### 2.8 *In Vivo* RAB1B Deficiency Exacerbates Liver Fibrosis by Regulating the Processes of Cuproptosis and Ferroptosis

**Figure 8:**
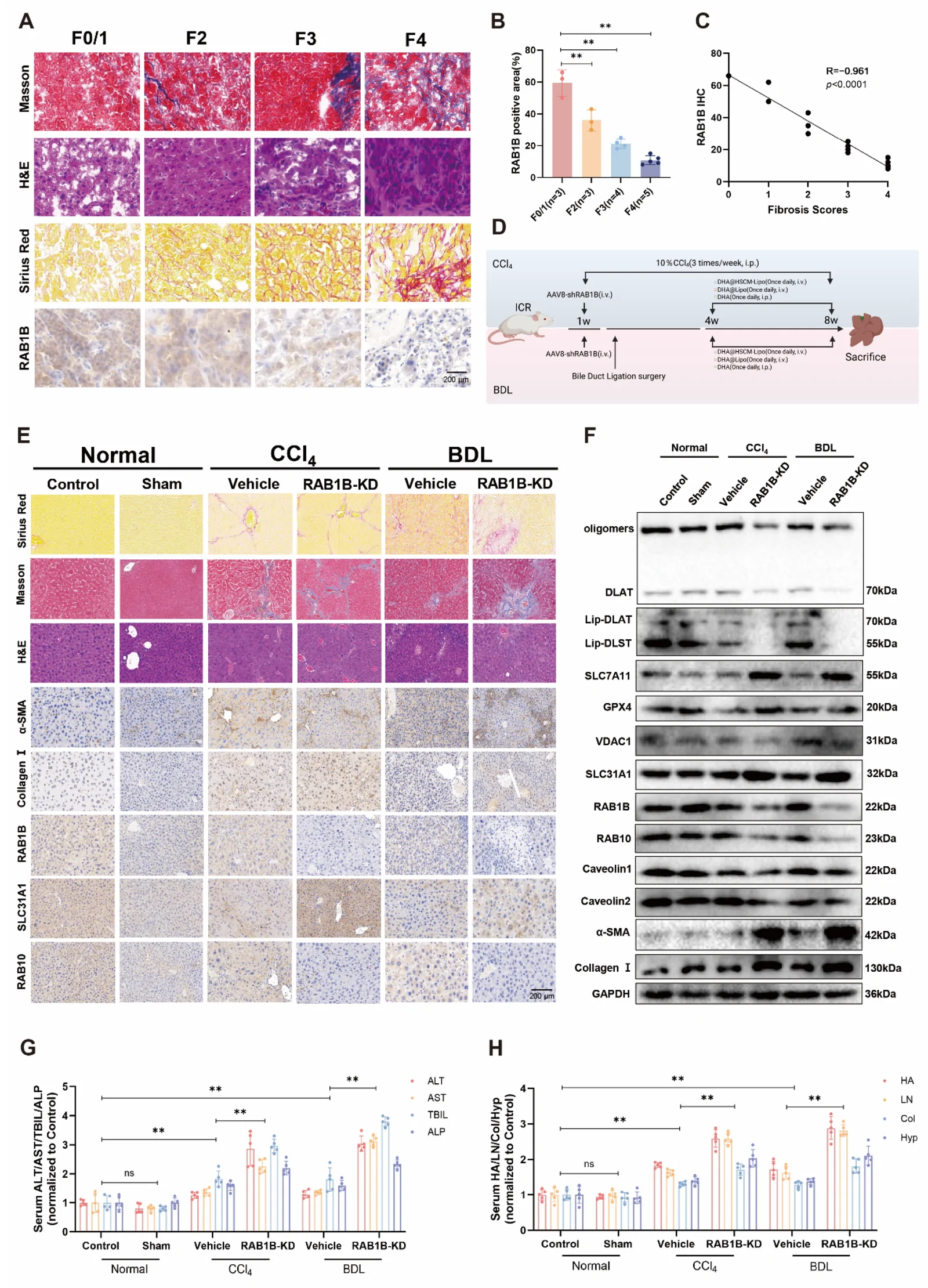
***In vivo* RAB1B deficiency exacerbates liver fibrosis by regulating the processes of cuproptosis and ferroptosis.** (A) Liver fibrosis staging was performed using the Ishak scoring system. Histopathological analysis was conducted on liver tissue samples from 15 human cases, including H&E staining, Masson's trichrome, Sirius Red staining, and IHC staining of RAB1B. Representative images are presented for the following fibrosis stages: F0/1 (3 cases), F2 (3 cases), F3 (4 cases), and F4 (5 cases). Scale bars: 200 μm, n = 6/group. (B) Quantification of RAB1B IHC-positive cells (n = 6/group). (C) Correlation analysis between RAB1B-IHC and liver fibrosis staging in human samples, including Spearman correlation coefficient (R) and p-value. (D) Experimental workflow for animal studies. (E) Liver tissues from normal controls, sham-operated, and CCl_4_/BDL-induced mouse liver fibrosis models (Vehicle and RAB1B-KD groups, n = 5) were subjected to IHC staining for α-SMA, Collagen I, RAB1B, SLC31A1, RAB10, and other markers. Scale bar: 200 μm. (F) Western blot analysis of α-SMA, Collagen I, RAB1B, SLC31A1, RAB10, VDAC1, SLC7A11, GPX4, Caveolin1, Caveolin2, Lip-DLST, Lip-DLAT, and oligomerized DLAT protein expression in liver tissues from normal (control and sham-operated) and liver fibrosis model mice (Vehicle and RAB1B-KD groups, n = 5). Protein expression was quantified by grayscale analysis (n = 3). (G) Serum analysis of ALT, AST, TBIL, and ALP levels in normal (control and sham-operated) and CCl_4_/BDL-induced liver fibrosis model mice (Vehicle and RAB1B-KD groups, n = 5). (H) Serum analysis of Col, Hyp, LN, and HA levels in normal (control and sham-operated) and CCl_4_/BDL-induced liver fibrosis model mice (Vehicle and RAB1B-KD groups, n = 5). Data are presented as mean ± SD, with p-values calculated using one-way ANOVA. ns, not significant; *p < 0.05, **p < 0.01.

To further investigate the relationship between RAB1B and the pathological progression of liver fibrosis, we analyzed RAB1B expression in human clinical liver fibrosis samples. Liver tissue sections from patients at different stages of fibrosis (F0/1, F2, F3, F4) were subjected to Masson staining, H&E staining, Sirius Red staining, and RAB1B immunohistochemistry (IHC). Typical features of fibrosis were observed in the liver samples (Figure 8A), while IHC results showed a decrease in RAB1B expression as the fibrosis stage progressed (Figure 8B). Semi-quantitative analysis of human liver clinical samples based on the Metavir score confirmed a significant negative correlation between RAB1B expression and the stage of liver fibrosis (Figure 8C). These results suggest that RAB1B may play a crucial inhibitory role in the onset and progression of liver fibrosis.

We further investigated the role of RAB1B in liver fibrosis using two commonly employed experimental models: carbon tetrachloride (CCl₄)-induced liver fibrosis and bile duct ligation (BDL). The schematic workflow of the *in vivo* experiments is presented in Figure 8D. Gross examination of liver morphology revealed varying degrees of fibrosis between the control, CCl₄, and BDL groups (Supplementary Fig. S8A). H&E staining, Masson staining, Sirius Red staining, and IHC for α-SMA and Collagen I revealed typical fibrosis features in the mouse liver (Figure 8E, Supplementary Fig. S8B). In both liver fibrosis models, liver RAB1B gene knockdown mediated by AAV8-RAB1B-KD significantly exacerbated the fibrosis process. IHC results showed a significant negative correlation between RAB1B expression and α-SMA (Figure 8E, Supplementary Fig. S8B).

Additionally, the downregulation of RAB1B was accompanied by decreased RAB10 expression and increased SLC31A1 expression (Figure 8E, Supplementary Fig. S8B), suggesting that RAB1B plays a key regulatory role in liver fibrosis by modulating copper ion vesicular transport and mediating cuproptosis. Further analysis confirmed that treatment with the AAV8 vector alone did not induce pathological changes in the liver of control mice, nor were there significant differences in the expression of RAB1B, α-SMA, Collagen I, RAB10, or SLC31A1 (Figure 8E, Supplementary Fig. S8B). To further determine the knockdown efficiency and cell-type specificity of AAV8-RAB1B-KD in distinct hepatic cell populations, five primary cell types were isolated from mouse livers. Western blotting revealed that RAB1B expression remained unchanged in pHeps, pKCs, pLSECs, and pBECs, whereas protein levels were significantly downregulated in pHSCs. These findings demonstrate that AAV8-RAB1B-KD mediates specific knockdown of RAB1B predominantly in HSCs, with minimal effects on other major parenchymal and non-parenchymal liver cells (Supplementary Fig. S8C).

In both models, RAB1B gene knockout significantly increased the liver weight-to-body weight ratio (Supplementary Fig. S8D), indicating that the knockout of RAB1B exacerbates liver enlargement. Further analysis revealed that, following RAB1B gene knockout, the expression of liver fibrosis markers α-SMA and Collagen I was significantly elevated (Figure 8F, Supplementary Fig. S8E). Additionally, the levels of DLAT oligomerized protein, acylated proteins, VDAC1, RAB10, Caveolin1, and Caveolin2 were significantly decreased, while the expression of SLC7A11, GPX4, and SLC31A1 was significantly increased (Figure 8F, Supplementary Fig. S8E). Moreover, measurements of liver injury markers (ALT, AST, ALP, and TBIL) and fibrosis markers (HA, LN, Hyp, and Col IV) confirmed that RAB1B gene knockout significantly accelerated liver fibrosis progression (Figure 8G-H).

In summary, RAB1B plays a crucial role in liver fibrosis progression by regulating copper ion vesicular endocytosis and the processes of cuproptosis and ferroptosis. These findings suggest that RAB1B could serve as a potential molecular target, offering new strategies for the treatment of liver fibrosis.

### 2.9 *In Vivo* DHA@HSCM-Lipo Alleviates Liver Fibrosis by Targeting RAB1B to Regulate Cuproptosis and Ferroptosis

**Figure 9:**
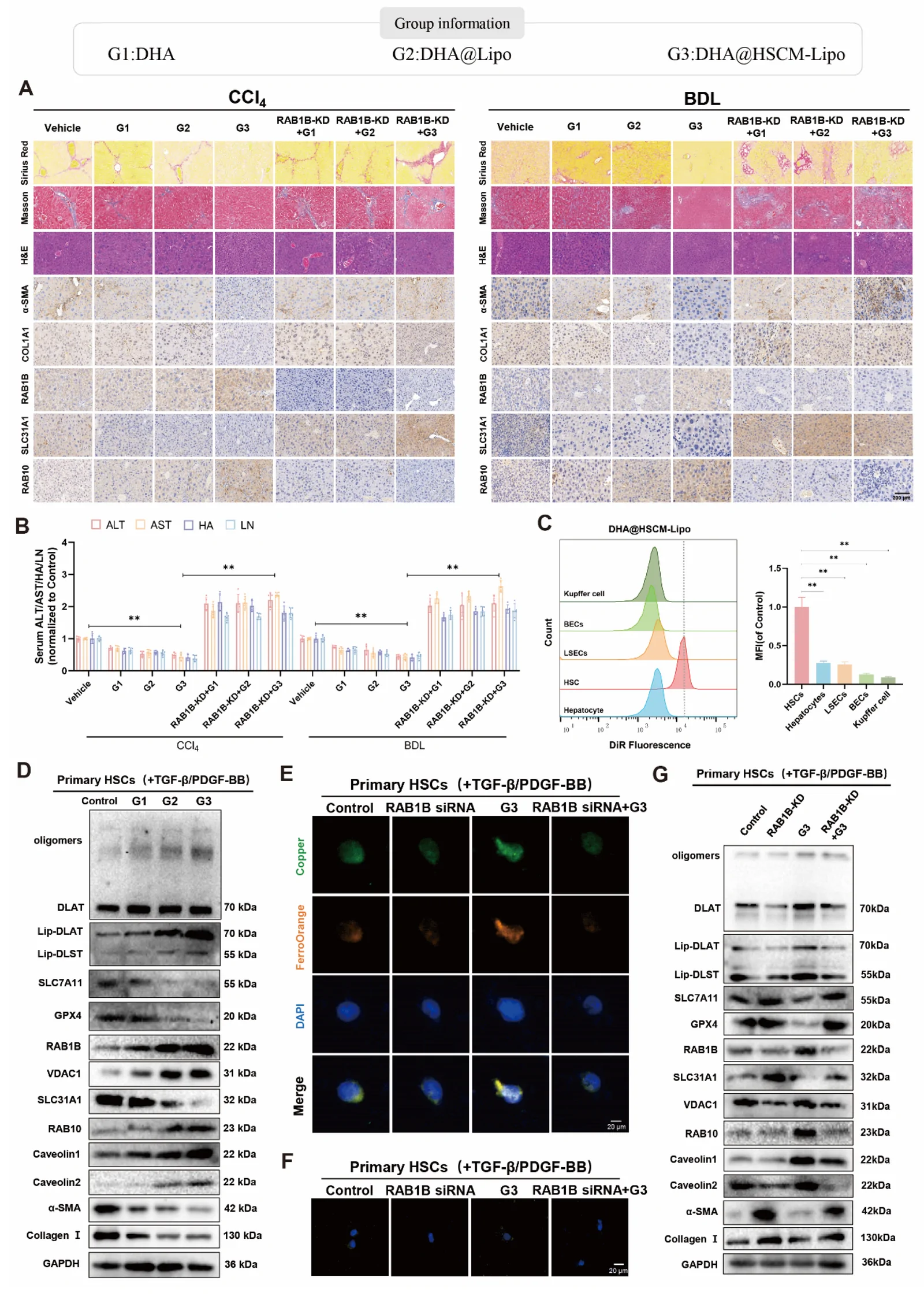
***In vivo* DHA@HSCM-Lipo alleviates liver fibrosis by targeting RAB1B to regulate cuproptosis and ferroptosis.** (A) Liver tissues from CCl_4_/BDL-induced mouse liver fibrosis models were subjected to H&E staining, Masson's trichrome, Sirius Red staining, and immunohistochemical analysis for α-SMA, Collagen I, RAB1B, RAB10, and SLC31A1 (n = 5). Scale bar: 200 μm. (B) Serum levels of liver injury markers (AST, ALT) and fibrosis markers (HA, LN) were assessed. (C) Flow cytometric analysis of DHA@HSCM-Lipo uptake efficiency. (D) Western blot analysis of α-SMA, Collagen I, SLC31A1, RAB1B, VDAC1, SLC7A11, GPX4, RAB10, Caveolin1, Caveolin2, Lip-DLST, Lip-DLAT, and oligomerized DLAT protein expression levels in primary HSCs treated with DHA (20 μM), DHA@Lipo, or DHA@HSCM-Lipo. Protein levels were quantified using grayscale analysis (n = 3). (E) Immunofluorescence analysis of copper and iron ion colocalization in primary HSCs treated with DHA@HSCM-Lipo, comparing control and RAB1B-KD groups (n = 3). Scale bar: 20 μm. (F) Immunofluorescence analysis of vesicle expression in primary HSCs treated with DHA@HSCM-Lipo, comparing control and RAB1B-KD groups (n = 3). Scale bar: 20 μm. (G) Western blot analysis of α-SMA, Collagen I, SLC31A1, RAB1B, VDAC1, SLC7A11, GPX4, RAB10, Caveolin1, Caveolin2, Lip-DLST, Lip-DLAT, and oligomerized DLAT protein levels in primary HSCs treated with DHA@HSCM-Lipo, comparing control and RAB1B-KD groups. Protein levels were quantified using grayscale analysis (n = 3). Data are presented as mean ± SD, with p-values calculated using one-way ANOVA. ns, not significant; *p < 0.05, **p < 0.01.

*In vitro* experiments demonstrated that DHA significantly inhibited HSCs activity by targeting RAB1B. To further validate the anti-fibrotic efficacy and underlying mechanisms *in vivo*, two distinct liver fibrosis models were established and treated with different drug delivery strategies. The experimental procedure is illustrated in Figure 8D. Gross observation of liver morphology is shown in Supplementary Fig. S9A. Immunohistochemistry revealed that DHA@HSCM-Lipo treatment upregulated RAB1B expression, whereas α-SMA and Collagen I levels were downregulated. H&E, Masson, and Sirius red staining further confirmed attenuated hepatic injury and reduced collagen deposition (Figure 9A, Supplementary Fig. S9B). Biochemical analysis indicated that levels of hepatic injury and fibrosis markers, including ALT, AST, HA, and LN, significantly decreased (Figure 9B). Concomitantly, the liver-to-body weight ratio was reduced (Supplementary Fig. S9C). These findings suggest that DHA@HSCM-Lipo effectively attenuates liver fibrosis. However, RAB1B knockdown significantly reversed these anti-fibrotic effects (Figure 9A, Supplementary Fig. S9B). In addition, pseudotime analysis revealed that RAB1B expression was downregulated during HSC activation (Supplementary Fig. S9D). These findings confirm that RAB1B serves as a critical target for DHA-mediated anti-fibrotic effects. To validate the targeting specificity of DHA@HSCM-Lipo, five primary cell types were co-incubated with DiR-labeled nanoparticles. Flow cytometry demonstrated that both the percentage of positive cells and mean fluorescence intensity (MFI) were significantly higher in pHSCs than in pHeps, pKCs, pLSECs, and pBECs (Figure 9C).

Subsequently, to validate the anti-fibrotic efficacy of DHA@HSCM-Lipo, primary HSCs were isolated and stimulated with TGF-β and PDGF-BB to recapitulate the hepatic injury and fibrogenic microenvironment. Molecular effects were then compared across different delivery modalities. The experimental results revealed that DHA@HSCM-Lipo showed the most pronounced changes in the expression of multiple key markers: DHA@HSCM-Lipo treatment significantly upregulated the expression of lipoylated proteins and DLAT oligomerized proteins while downregulating SLC7A11 and GPX4 expression, thereby promoting cuproptosis and ferroptosis (Figure 9D, Supplementary Fig. S9F). Furthermore, DHA@HSCM-Lipo treatment concurrently upregulated RAB1B, RAB10, VDAC1, and Caveolin 1/2, proteins associated with copper-ion vesicular endocytosis, and concomitantly suppressed α-SMA and Collagen I expression. These findings confirm that DHA@HSCM-Lipo exerts anti-fibrotic effects by enhancing membrane trafficking and mitochondrial function (Figure 9D, Supplementary Fig. S9F). Compared to unmodified liposome particles, cell membrane-coated liposomes typically show longer circulation times in the bloodstream. To evaluate *in vivo* accumulation efficiency, hepatic drug concentrations were determined in mice from each group. Results showed that the DHA@HSCM-Lipo group exhibited significantly elevated accumulation (Supplementary Fig. S9E). These findings indicate that HSC membrane biomimetic modification confers a superior delivery strategy by prolonging circulation time and enhancing target organ accumulation for anti-fibrotic therapy.

Based on these findings, the molecular effects of DHA@HSCM-Lipo in HSCs and its regulation of fibrogenic signaling pathways were further investigated. Immunofluorescence revealed that DHA@HSCM-Lipo treatment significantly increased copper and iron ion levels (Figure 9E, Supplementary Fig. S9G). Furthermore, vesicle formation was markedly increased (Figure 9F). These observations suggest that these effects are closely associated with RAB1B upregulation. Western blotting further confirmed these results: DHA@HSCM-Lipo treatment upregulated the expression of lipoylated proteins, DLAT oligomerized proteins, RAB1B, RAB10, VDAC1, Caveolin1, and Caveolin2, while downregulating the expression of SLC7A11, GPX4, SLC31A1, α-SMA, and Collagen I. This promoted cuproptosis and ferroptosis in HSCs and facilitated the endocytosis and transport of copper ion vesicles, effectively alleviating liver fibrosis (Figure 9G, Supplementary Fig. S9H). However, RAB1B silencing significantly reversed these restorative effects, confirming that RAB1B serves as the key effector molecule of this nanosystem (Figure 9G, Supplementary Fig. S9H).

*In vivo* experiments demonstrated that DHA@HSCM-Lipo specifically recognized HSCs in the liver, enhanced hepatic accumulation, and significantly attenuated collagen deposition and liver injury. Its therapeutic efficacy was dependent on RAB1B-mediated activation of cuproptosis-ferroptosis. Knockdown of RAB1B substantially compromised the anti-fibrotic effects of this nanosystem, confirming RAB1B as a critical target for *in vivo* action, while simultaneously validating the targeting mechanism and therapeutic efficacy of this biomimetic delivery system.

## 3. Discussion

DHA, a pivotal derivative of artemisinin, exhibits enhanced bioactivity and superior pharmacokinetic properties. In recent years, accumulating evidence has demonstrated that beyond its antimalarial efficacy, DHA possesses extensive therapeutic potential in antitumor, anti-inflammatory, and antiviral applications^47^, ^48^. Our previous study revealed that DHA exerts anti-fibrotic effects by promoting NR1D1-mediated RAB7 ubiquitination to regulate lipophagy in HSCs. Based on these findings, this study further elucidates the mechanisms by which DHA modulates metal ion-dependent cell death to combat liver fibrosis. This study also demonstrated that DHA triggers a sequential cascade of cuproptosis and ferroptosis, thereby suppressing HSC proliferation and activation to exert anti-fibrotic effects.

The interplay among distinct cell death modalities represents a frontier in cell fate determination; however, whether these modalities constitute a regulatable cascade, and the associated pathophysiological significance, remain poorly defined. The central finding of this study is that DHA temporally activates cuproptosis and ferroptosis, thereby suppressing HSCs activation. Mitochondrial dysfunction and iron-sulfur cluster protein degradation induced by cuproptosis do not represent terminal events but rather serve as upstream signals triggering ferroptosis. This finding challenges the prevailing notion that cuproptosis and ferroptosis operate independently, thereby expanding the regulatory network of metal ion-dependent cell death. Combined targeting of cuproptosis initiation and ferroptosis amplification may constitute a promising therapeutic strategy against liver fibrosis.

During this process, mitochondria function as the core hub integrating cuproptosis and ferroptosis, converting initial copper ion stress into irreversible death signals through a positive feedback loop. This study identified VDAC1 as the key effector molecule mediating DHA-induced signal transduction. DHA enhances VDAC1-mediated mitochondrial permeability, driving metal ion accumulation and oxidative stress to promote cell death. Protein-protein interaction screening further established RAB1B as a critical upstream target governing mitochondrial dysfunction. Furthermore, AFM analysis demonstrated that DHA treatment reduces plasma membrane elastic modulus, concomitant with a marked increase in nanoscale surface invaginations. This morphological feature suggests that DHA may activate specific endocytic programs through remodeling of the membrane mechanical microenvironment. Notably, RAB1B is conventionally recognized as a molecular switch regulating vesicular trafficking; the present findings indicate that it may additionally function as a mechanosensor. Mechanosignals generated by enhanced endocytosis are transmitted via RAB1B to VDAC1, promoting vesicle-mitochondria docking and copper ion transmembrane transport. Optical tweezer experiments and truncation analysis confirmed that the N-terminus (amino acids 1-80) of RAB1B constitutes the critical mechanoresponsive domain. Molecular dynamics simulations further revealed hydrogen bond and salt bridge networks at the binding interface, providing the structural basis for understanding mechanochemical signal transduction.

This process also elucidates a temporal switching mechanism by which cells adapt to copper overload: when classical copper transport mediated by SLC31A1 is compromised due to ubiquitin-dependent degradation, the RAB10-driven vesicular endocytic pathway establishes compensatory uptake. The molecular switch governing this transition lies in rapid post-translational modification. This study demonstrated that KAT2A-mediated succinylation of USP11 suppresses its deubiquitinase activity, thereby accelerating proteasomal degradation of SLC31A1 and shifting cellular copper transport from canonical membrane transport to vesicular endocytosis. This paradigm may extend to the metabolic regulation of other metal ions, offering insights into stress-induced remodeling of metal ion homeostasis.

In recent years, liposomes have emerged as a focal point of research in drug delivery due to their excellent biocompatibility, stability, and targeting capabilities^49^. However, conventional liposomes still face certain limitations in targeting efficiency and drug accumulation. To overcome these challenges, researchers have actively explored surface modifications of liposomes to enhance their targeting ability and delivery efficiency^50^-^53^. This study developed an HSC membrane-biomimetic liposomal delivery system, DHA@HSCM-Lipo, which integrates three functional modules: CD47-mediated immune evasion, FNIII10-directed HSC homing, and ApoE-dependent hepatic tropism, thereby achieving efficient nanoparticle delivery. *In vivo* experiments demonstrated that, compared with unmodified liposomes, DHA@HSCM-Lipo significantly elevated hepatic drug concentrations, reduced collagen deposition, attenuated hepatic injury, and exhibited superior therapeutic efficacy in promoting vesicular endocytosis and transport as well as activating the cuproptosis-ferroptosis cascade. These findings indicate that DHA@HSCM-Lipo improves drug delivery efficiency and provides a targeted therapeutic strategy for liver fibrosis.

In summary, this study innovatively elucidates the multi-layered mechanism by which DHA regulates RAB1B to promote copper ion vesicular endocytosis via SLC31A1 degradation under copper overload, thereby sequentially activating cuproptosis and ferroptosis. The HSC membrane-biomimetic liposome delivery system, DHA@HSCM-Lipo, constructed based on this mechanism significantly enhances targeted delivery and therapeutic efficacy. This strategy provides new insights for the treatment of metal ion-related diseases and precise drug delivery.

## 4. Materials and Methods

### 4.1 Clinical Samples

In this study, liver tissue specimens were collected from patients at the Nanjing University of Chinese Medicine Affiliated Nanjing Hospital of Traditional Chinese Medicine. All patients provided informed consent, and the research protocol was approved by the Medical Ethics Committee of Nanjing University of Chinese Medicine Affiliated Nanjing Hospital of Traditional Chinese Medicine. This study adhered to the principles outlined in the Declaration of Helsinki. Tissue specimens were embedded in paraffin, sectioned, and subjected to histological examination. According to the METAVIR scoring system, histological fibrosis staging was classified as no or mild fibrosis (F0/1), moderate fibrosis (F2), severe fibrosis (F3/F4), and cirrhosis (F4). Detailed patient characteristics can be found in previous studies^54^.

### 4.2 Animals and Grouping

The animal experiments were conducted at the Animal Experiment Center of Nanjing University of Chinese Medicine, with approval from the Nanjing University of Chinese Medicine Ethics Committee (Approval No.: 202504A092). Six-week-old male SPF-grade Institute of Cancer Research (ICR) mice, weighing approximately 20 g, were purchased from Jiangsu Qinglongshan Biotechnology Co., Ltd. Mice were housed in individually ventilated cages (IVC) with ad libitum access to food and water. The AAV8-ssAAV.TBG669.GFAP.mRAB1B_miR30 viral vector (1E+13 GC mL⁻¹) for hepatic mRab1B knockdown was provided by PackGene Biotech and administered via tail vein injection. Liver fibrosis was induced in mice by intraperitoneal CCl₄ injection or bile duct ligation (BDL). Detailed modeling procedures have been described in previous publications^42^. In this experiment, after one week of acclimatization, the mice were randomly assigned to groups (n = 5 per group): Normal group, including Normal control group, normal control group + empty plasmid group. BDL model group, including Sham operation group, BDL + empty plasmid group, BDL + DHA group, BDL + DHA@Lipo group, BDL + DHA@HSCM-Lipo group, BDL + Rab1BshRNA group, BDL + Rab1BshRNA + DHA group, BDL + Rab1BshRNA + DHA@Lipo group, BDL + Rab1BshRNA + DHA@HSCM-Lipo group. CCl_4_ model group: CCl_4_ + empty plasmid group, CCl_4_ + DHA group, CCl_4_ + DHA@Lipo group, CCl_4_ + DHA@HSCM-Lipo group, CCl_4_ + Rab1BshRNA group, CCl_4_ + Rab1BshRNA + DHA group, CCl_4_ + Rab1BshRNA + DHA@Lipo group, CCl_4_ + Rab1BshRNA + DHA@HSCM-Lipo group. Following establishment of the liver fibrosis model, mice received tail vein injections every two days for four weeks. At the end of the experiment, euthanasia was performed according to animal ethics guidelines, and tissues were collected for further analysis.

### 4.3 Culture Conditions for Primary Cells and Cell Lines

LX2 cells and RAW264.7 (Shanghai Cell Bank, Chinese Academy of Sciences) were cultured in high-glucose DMEM (HyClone, Cytiva, Cat# SV30303.01) supplemented with 10% fetal bovine serum (Cellmax, SA301.01, Beijing) and 1% penicillin-streptomycin (Gibco, USA). THLE-2 cells were maintained in THLE-2 complete medium (Wuhan Procell Biotechnology Co., Ltd.). All cells were incubated at 37 °C in a humidified incubator with 5% CO₂. Cell line authentication was performed for both THLE-2 and LX2 cells. The isolation and culture procedure of primary cells is detailed in previous studies^42^.

### 4.4 scRNA-seq Analysis

The data used in this study were obtained from the GEO database (GSE210077), which includes healthy liver tissue samples as well as liver tissue samples with varying degrees of fibrosis. The raw data files were processed into the 10X Genomics input format. Data were read using Seurat (4.4.0), with preliminary quality control performed based on the following criteria: nFeature_RNA > 200, nFeature_RNA < 8000, and percent.MT < 10. This filtering reduced the initial 70,520 cells to 62,542 cells. Doublet prediction was performed using scDblFinder (v1.16.0), and 5,690 doublets were subsequently removed. Harmony (v1.2.1) was then applied for batch effect correction to enhance clustering consistency across samples. Principal component analysis (PCA) was performed using the RunPCA function in Seurat (4.4.0), followed by cell clustering with the FindNeighbors and FindClusters functions. The optimal clustering resolution was determined using the clustree (version 0.5.1) method. Data visualization was performed using Uniform Manifold Approximation and Projection (UMAP) and t-distributed Stochastic Neighbor Embedding (t-SNE). Cuproptosis and Copper homeostasis scores were calculated using the AUCell algorithm from the irGSEA (3.3.2) tool. The Cuproptosis score was based on the gene set ('FDX1', 'LIAS', 'LIPT1', 'DLAT', 'PDHA1', 'PDHB', 'DLST'), and the Copper homeostasis score was based on the gene set ('SLC31A1', 'ATP7A', 'ATP7B', 'ATOX1', 'SOD1', 'MT1', 'MT2'). Following annotation and isolation of HSC subpopulations, pseudotime analysis was performed using Slingshot (v2.7.0), and results were visualized with SCP (v0.5.6).

### 4.5 CCK-8 Cell Viability Assay

LX2 cells were seeded into 96-well plates at a density of 5,000 cells per well. Following adherence, cells were treated with the indicated concentrations of drugs for 24 h. Subsequently, 10 μL of CCK-8 reagent (#E-CK-A362, Elabscience Biotechnology Co., Ltd.) was added to each well, and plates were incubated at 37 °C in a humidified incubator with 5% CO₂ for 1 h. Absorbance at 450 nm was measured using a microplate reader, and cell viability was calculated for each concentration. Cell viability (%) was determined according to the following formula: (OD experimental - OD blank) / (OD control - OD blank) × 100%.

### 4.6 ICP-MS Measurement of Copper and Iron Ion Content

Intracellular copper and iron ion levels were determined by inductively coupled plasma tandem mass spectrometry (ICP-MS/MS; iCAP TQ, Thermo Fisher Scientific, USA). Cell samples were subjected to microwave-assisted digestion with nitric acid and hydrogen peroxide. Quantification was performed using ⁶³Cu and ⁵⁷Fe as analytical isotopes with an internal standard method. Ion concentrations were calculated by standard curve calibration.

### 4.7 Measurement of Glutathione, 4-HNE, and MDA Levels

The intracellular malondialdehyde (MDA) content was determined using the MDA Detection Kit (Best Bio; #BB4709). The 4-HNE content was measured using a 4-HNE ELISA Kit (YIFEIXUE Biotech; #YFXEH00631). Glutathione (GSH) levels in LX2 cells were measured using the Glutathione Assay Kit (Best Bio; #BB4711). All assays were performed according to the manufacturer's protocols. Absorbance was measured at the indicated wavelengths using a microplate reader, and concentrations were determined by standard curve calibration.

### 4.8 Mitochondrial Extraction

Following 24 h of drug treatment, cells were washed twice with ice-cold PBS on ice. After collection, cells were lysed in lysis buffer supplemented with phenylmethylsulfonyl fluoride (PMSF) and phosphatase inhibitor solution (PS). The lysates were incubated on ice for 10 min, and cell disruption was facilitated by sonication. After cell disruption, the mixture was centrifuged at low speed (4 °C, 1,000 × g, 10 minutes) to remove cell nuclei and unbroken cells, and the supernatant was collected. The supernatant was then subjected to high-speed centrifugation (4 °C, 15,000 × g, 25 minutes) to pellet the mitochondria, and the supernatant was discarded. The mitochondrial pellet was resuspended in pre-chilled lysis buffer, and further purified by high-speed centrifugation (4 °C, 15,000 × g, 20 minutes) to remove cellular debris and impurities, resulting in the final mitochondrial preparation. Mitochondrial purity was assessed by Western blotting for the mitochondrial marker TOM20.

### 4.9 Quantitative Real-Time Polymerase Chain Reaction (qRT‒PCR)

Total RNA was extracted from LX2 cells using TRIzol reagent. RNA concentration was measured using a NanoDrop 2000 spectrophotometer. Total RNA (1 μg) was reverse-transcribed into cDNA using the HiFiScript All-in-one RT Master Mix with gDNA remover (CW3371, CABIO, China) according to the manufacturer's protocol. The resulting cDNA was diluted 10-fold and used as the template for qPCR. qPCR was performed on a QuantStudio 6 Flex Real-Time PCR System (Thermo Fisher Scientific, USA) using SYBR Green dye (SuperStar Universal SYBR Master, CW3360, CWBIO, China). The thermal cycling conditions consisted of an initial denaturation at 95 °C for 3 min, followed by 40 cycles of 95 °C for 5 s and 60 °C for 30 s. GAPDH served as the internal reference gene, and relative expression levels of target genes were calculated using the 2^(-ΔΔCt) method.

### 4.10 Western Blot Analysis

Following 24 h of drug treatment, the culture medium was aspirated, and cells were washed twice with ice-cold PBS. Cells were lysed in RIPA buffer supplemented with 1 mM PMSF, 1× phosphatase inhibitor cocktail, and 1× protease inhibitor cocktail on ice for 30 min, with vortexing at 10 s intervals every 5 min. Lysates were centrifuged at 12,000 × g for 15 min at 4 °C, and supernatants were collected. Total protein concentrations were determined using a BCA assay kit. Equal amounts of protein were mixed with 5× loading buffer and denatured at 95 °C for 5 min. The separating and stacking gels were prepared using 30% (29:1) acrylamide/bis-acrylamide stock solution (Solarbio, A1010) and 4× SDS-PAGE separating/stacking gel buffers (Solarbio, S1051 and S1052). Protein samples were separated by sodium dodecyl sulfate-polyacrylamide gel electrophoresis (SDS-PAGE) through a stacking gel at 90 V for 20 min and a resolving gel at 120 V for 120 min. Proteins were then transferred onto 0.2 μm PVDF membranes at 110 V for 120 min. Membranes were blocked with 5% skim milk at room temperature for 2 h to prevent non-specific binding. Subsequently, membranes were cut according to the molecular weights of target proteins and incubated with corresponding primary antibodies at 4 °C overnight. After three washes with TBST (10 min each), membranes were incubated with HRP-conjugated secondary antibodies (1:5,000) at room temperature for 2 h. Following additional washes with TBST, bands were visualized using enhanced chemiluminescence reagent (SuperKine™ West Femto Maximum Sensitivity Substrate, BMU102, Abbkine) and exposed in a chemiluminescence gel imaging system. Densitometric quantification was performed using Image Lab software, and relative expression levels were normalized to GAPDH.

### 4.11 siRNA, Overexpression Plasmid, and Site-Directed Mutagenesis Plasmid Transfection

Cells were seeded into six-well plates and cultured until reaching 30%-50% confluence prior to transfection. Transfection was performed according to the manufacturer's protocol. Briefly, siRNA was diluted in Opti-MEM reduced-serum medium, and SuperKine™ Lipo3.0 transfection reagent (Abbkine, China) was mixed with Opti-MEM at the recommended ratio. The two solutions were combined and incubated at room temperature for 10-15 min to permit siRNA-lipid complex formation. Concurrently, the culture medium was replaced with serum-free medium, and the transfection complexes were then added to the cell culture system with gentle agitation. The medium was replaced with complete medium at 24 h post-transfection, and cells were harvested at 72 h for subsequent experiments. For transfection of overexpression and site-directed mutagenesis plasmids, plasmid DNA and transfection reagent were diluted and complexed at a mass ratio of 1:2.5 (μg:μL), with the remaining steps performed as described above.

### 4.12 TEM Characterization

Cell samples were fixed in 2.5% glutaraldehyde and 4% paraformaldehyde mixed fixative at 4 °C for 4 h, followed by three washes with 0.1 M PBS. Post-fixation was performed with 1% osmium tetroxide for 1 h. Samples were then dehydrated through a graded ethanol series (30%, 50%, 70%, 80%, 90%, and 100%, 15 min each), transitioned through acetone, and embedded in Epon 812 resin. Ultrathin sections (80 nm) were prepared using an ultramicrotome, stained with uranyl acetate and lead citrate, and examined under a JEOL JEM-2100 transmission electron microscope at an accelerating voltage of 200 kV. Images were acquired with a Gatan CCD camera and processed using DigitalMicrograph software.

### 4.13 Immunofluorescence

Cells were seeded onto coverslips in 24-well plates at a density of 2 × 10⁴ cells/mL and cultured to appropriate confluence prior to drug treatment. Cells were fixed with 4% paraformaldehyde at room temperature for 30 min, washed three times with PBS, and permeabilized with 0.5% Triton X-100 at room temperature for 15 min. Following additional washes with PBS, cells were blocked with 3% bovine serum albumin (BSA) at room temperature for 2 h. After removal of the blocking solution, cells were incubated with corresponding primary antibodies at 4 °C overnight. The following day, cells were washed three times with PBS (5 min each) and incubated with fluorophore-conjugated secondary antibodies (1:500) at room temperature for 2 h in the dark. Nuclei were counterstained with DAPI (Keygen BioTECH, China) at room temperature for 5 min, and slides were mounted with antifade mounting medium. Images were acquired under a laser scanning confocal microscope at the indicated excitation wavelengths to analyze the distribution and expression of target proteins or molecules.

### 4.14 AFM Measurement of Cell Surface Force Curves

LX2 cells were seeded onto poly-L-lysine-coated coverslips and cultured to 70-80% confluence at 37 °C in a humidified incubator with 5% CO₂. The culture medium was aspirated, and cells were gently washed twice with pre-warmed PBS. HEPES buffer (10 mM HEPES, 150 mM NaCl, 5 mM KCl, 1 mM CaCl₂, 1 mM MgCl₂, pH 7.4) was then applied to maintain cell viability. Force spectroscopy was performed using an atomic force microscope (Bruker Dimension Icon) in PeakForce QNM mode. Silicon nitride probes with a spring constant of 0.5 N/m and a tip radius of 2 nm were employed for scanning. The peripheral flattened regions of LX2 cells (10 × 10 μm², excluding the nucleus) were selected for imaging. Force-distance curves were acquired at distinct locations on the cell surface. Young's modulus was calculated by fitting the force-distance curves to the Hertz-Sneddon model to reflect plasma membrane stiffness.

### 4.15 Optical Tweezers Integrated with Confocal Fluorescence Microscopy

The GFP-tagged RAB1B expression plasmid and mCherry-tagged VDAC1 expression plasmid were transfected into LX2 cells. After transfection, the cells were cultured at 37 °C in a 5% CO₂ atmosphere for 24 hours to ensure adequate expression of the fluorescent fusion proteins. Prior to experiments, carboxylated polystyrene microspheres (3 μm in diameter) were sonicated in buffer containing 0.5 mg/mL BSA and set aside. A confocal optical tweezers system equipped with a 1064 nm near-infrared trapping laser and a 100× water immersion objective was employed on a temperature-controlled stage (37°C, 5% CO₂). The optical trap position was controlled using an acousto-optic deflector (AOD). After trapping a PS microsphere, the trapping laser power at the sample plane was calibrated to 20 mW. The trapped microsphere was precisely positioned in close proximity to the target cell membrane, and the laser power was adjusted to maintain stable interaction between the microsphere and the membrane surface. During experiments, GFP and mCherry were sequentially excited with 488 nm and 561 nm laser lines, respectively. Dual-channel fluorescence images were acquired synchronously using a high-sensitivity detector to monitor the localization dynamics of RAB1B and VDAC1 and their responses to mechanical stimulation in real time.

### 4.16 Molecular Docking

The binding mode between RAB1B and VDAC1 proteins was predicted using HDOCKlite. The structural files of these proteins were obtained from the PDB database. These structures were then processed with PyMOL 2.5.3, including the removal of non-protein components. The docking study was performed using the default settings of HDOCK for global rigid-body docking. After docking, the binding mode with the best binding energy was selected and visualized using PyMOL 2.5.3.

### 4.17 Molecular Dynamics Simulation

The protein-protein complex obtained from docking was used as the initial structure for all-atom molecular dynamics (MD) simulations, which were performed using Amber 22 software. Prior to simulation, the system underwent energy minimization, including 2500 steps of steepest descent and 2500 steps of conjugate gradient methods. After energy optimization, the system was gradually heated from 0 K to 298.15 K over 200 ps at constant volume and a constant heating rate. Following temperature equilibration at 298.15 K, a 500 ps NVT simulation was performed to evenly distribute solvent molecules in the solvent box. Finally, a 500 ps NPT simulation was conducted for system equilibration under constant pressure. The entire system was subjected to a 100 ns NPT simulation under periodic boundary conditions. The nonbonded cutoff distance was set to 10 Å, and the Particle Mesh Ewald (PME) method was used to calculate long-range electrostatic interactions. The SHAKE method was applied to constrain bond lengths involving hydrogen atoms, and the Langevin algorithm was used for temperature control with a collision frequency of γ = 2 ps⁻¹. The system pressure was maintained at 1 atm, and an integration step size of 2 fs was used, with trajectory data saved every 10 ps for subsequent analysis.

### 4.18 Isolation of HSC Membranes

HSC membranes were extracted as previously described^55^. When HSCs reached 90% confluence in culture, they were washed twice with cold PBS and collected in cold PBS. Cells were pelleted by centrifugation at 1,000 × g for 5 min at 4 °C and resuspended in 1 mL hypotonic buffer (225 mM mannitol, 75 mM sucrose, 30 mM Tris-HCl, pH 7.4, 0.5% BSA, 0.5 mM EGTA, and protease inhibitor cocktail). Cells were then disrupted by sonication, and the homogenate was centrifuged at 3,000 × g for 5 min at 4 °C to remove nuclei and unbroken cells. The supernatant was collected and centrifuged at 10,000 × g for 10 min at 4 °C to eliminate mitochondria and other organelles. The resulting supernatant was subsequently subjected to ultracentrifugation at 100,000 × g for 2 h at 4 °C. The pellet, representing the membrane fraction, was resuspended in a minimal volume of PBS. Protein concentrations of the HSC membrane fraction were determined by the BCA assay, and aliquots were stored at -80 °C until use.

### 4.19 Preparation of DHA@Lipo

Dipalmitoylphosphatidylcholine (DPPC), dipalmitoylphosphatidylglycerol (DPPG), 1,2-distearoyl-sn-glycero-3-phosphoethanolamine-N-[methoxy(polyethylene glycol)-2000] (DSPE-PEG2000), and cholesterol (CHOL) were dissolved in chloroform at a molar ratio of 10:3:3:2. All reagents were purchased from AVT (Shanghai) Pharmaceutical Technology Co., Ltd. DHA was added at a molar ratio of 10:1 (total phospholipid to drug), and the mixture was thoroughly homogenized. Organic solvents were removed by rotary evaporation to form a uniform lipid film on the flask wall. The lipid film was hydrated with an appropriate volume of PBS in an ultrasonic bath to generate a liposomal suspension. Finally, unencapsulated drug was removed by dialysis against PBS overnight using a 1 kDa dialysis membrane. The resulting DHA@Lipo was filtered through a 0.22 μm membrane and stored at 4 °C until use.

### 4.20 Preparation of DHA@HSCM-Lipo

Extracted HSC membranes were fused with DHA@Lipo at a mass ratio of 10:1 (total phospholipid to membrane protein). The membrane protein concentration was determined by the BCA assay to calculate the required membrane input. The mixture was extruded through a polycarbonate membrane with a pore size of 200 nm using a mini-extruder for 12 cycles to ensure uniform membrane fusion and particle size homogenization. The extrudate was centrifuged at 15,000 × g for 30 min at 4 °C to remove unfused membrane debris. The resulting supernatant was collected as DHA@HSCM-Lipo.

### 4.21 Characterization of DHA@HSCM-Lipo

Transmission electron microscopy (TEM, HT7700, HITACHI, Japan) was used to capture the TEM images of DHA@HSCM-Lipo. The liposomes were dispersed in PBS, and their hydrodynamic diameter and Zeta potential were measured using a nanoparticle size and Zeta potential analyzer (Nano ZS, Malvern, UK). Atomic force microscopy (Dimension FastScan, Bruker, USA) was employed to assess the surface morphology and uniformity. The encapsulation efficiency and drug loading of DHA in the liposomes were determined using high-performance liquid chromatography (HPLC, Shimadzu, Kyoto, Japan). The functional group structure of DHA@HSCM-Lipo was characterized using Fourier-transform infrared (FTIR) spectroscopy (ANTARIS, Thermo Fisher, USA).

The drug encapsulation efficiency and drug loading efficiency were calculated using the following formulas:

Encapsulation Efficiency (%) = Mass of drug encapsulated in nanoparticles / Initial mass of drug × 100%

Loading Efficiency (%) = Mass of drug encapsulated in nanoparticles / (Total mass of nanoparticles) × 100%

### 4.22 Protein Characterization of DHA@HSCM-Lipo

The protein profiles of DHA@Lipo, HSC membranes, and DHA@HSCM-Lipo were analyzed by SDS-PAGE. HSC membranes or nanoparticles were resuspended in RIPA buffer supplemented with protease inhibitors and lysed on ice for 30 min. Lysates were centrifuged at 12,000 × g for 15 min at 4 °C, and supernatants were collected. Protein concentrations were determined by the BCA assay. Samples were mixed with 5× loading buffer and denatured at 95 °C for 10 min. Equal amounts of protein were separated by 10% SDS-PAGE. Gels were stained with Coomassie brilliant blue rapid staining solution (E901-01, Vazyme, Nanjing, China) and visualized using a gel imaging system.

### 4.23 Co-Immunoprecipitation

The specific experimental procedure has been described in previous publications^56^.

### 4.24 Cellular Thermal Shift Assay

Following treatment with DHA or DMSO (vehicle control), LX2 cells were harvested and lysed. The lysate was divided into eight aliquots and heated at 43, 46, 49, 52, 58, or 61 °C for 3 min. Samples were subsequently snap-frozen in liquid nitrogen and thawed at room temperature. This freezing-thawing cycle was repeated twice to ensure complete cell lysis. The cell lysates were then centrifuged at 4 °C, 16,000 × g for 20 minutes, and the supernatants were collected for protein denaturation analysis. The denatured proteins were analyzed by Western blot to assess RAB1B levels, and a melting curve was plotted for comparison.

### 4.25 Co-immunoprecipitation and Mass Spectrometry Analysis

The specific experimental procedure has been described in previous publications^42^.

### 4.26 Protein Affinity Purification

The human RAB1B gene was cloned into the pET-28a (+) vector to construct an N-terminal His-tagged fusion expression plasmid, which was subsequently transformed into E. coli BL21(DE3) competent cells. Isopropyl β-D-1-thiogalactopyranoside (IPTG) was added to a final concentration of 0.5 mM when the bacterial culture reached an optical density at 600 nm (OD₆₀₀) of 0.6-0.8, followed by induction at 18 °C for 16 h. Cells were harvested, resuspended in lysis buffer, and disrupted by sonication (200 W, 3 son / 5 s off, total duration 20 min). The lysate was centrifuged at 12,000 × g for 30 min at 4 °C, and the supernatant was collected. Protein purification was performed by Ni-NTA affinity chromatography. The column was equilibrated with binding buffer (50 mM Tris-HCl, pH 8.0, 300 mM NaCl, 10 mM imidazole, 1 mM PMSF). Following sample loading, bound proteins were eluted with a stepwise imidazole gradient (20, 50, 100, and 250 mM) in elution buffer, and fractions were collected. Peak fractions containing the target protein were pooled and dialyzed against 20 mM HEPES, pH 7.4, 150 mM NaCl, 10% glycerol, and 1 mM dithiothreitol (DTT) at 4 °C using a 10 kDa dialysis membrane, with three buffer exchanges at 4 h intervals. The dialyzed protein was concentrated by ultrafiltration, aliquoted, and stored at -80 °C. Purity was assessed by SDS-PAGE and confirmed by Western blotting.

### 4.27 SPR Affinity Assay

SPR technology was employed to detect the binding affinity between DHA and RAB1B protein. RAB1B protein was immobilized onto the surface of a sensor chip via amine coupling. HBS-EP+ buffer (10 mM HEPES, pH 7.4, 150 mM NaCl, 3 mM EDTA, 0.05% v/v Surfactant P20) containing 2% DMSO served as both the running buffer and sample diluent. DHA was dissolved in running buffer to prepare serial dilutions (5, 10, 20, 40, 80, and 160 μM) and injected sequentially into the chip channel from low to high concentrations. The chip surface was regenerated with 10 mM glycine-HCl, pH 2.0, following each cycle. A blank-coupled channel was employed as reference, and an equivalent volume of DMSO-containing running buffer was used as a negative control. Binding and dissociation were monitored in real time at 25 °C. Sensorgrams were fitted to a 1:1 Langmuir binding model using kinetic analysis software to determine the binding kinetics and affinity of the DHA-RAB1B interaction.

### 4.28 Hematoxylin and Eosin (H&E) Staining

Samples were fixed in 4% paraformaldehyde for 48 hours and then dehydrated in 70% ethanol for 12 hours. The tissues were trimmed to appropriate sizes (0.5 cm × 0.5 cm × 0.5 cm), further dehydrated, and embedded in paraffin. Paraffin-embedded tissue samples were sectioned, baked, and deparaffinized. Afterward, the sections were stained with hematoxylin and eosin (H&E), air-dried, and mounted with coverslips. Finally, six random fields were selected and observed under a microscope for imaging.

### 4.29 Immunohistochemistry (IHC)

Fresh tissue blocks were fixed in 4% paraformaldehyde, dehydrated through graded ethanol, cleared with xylene, and embedded in paraffin. After deparaffinization, antigen retrieval, and blocking of nonspecific binding sites, primary and secondary antibodies were applied sequentially. 3,3'-Diaminobenzidine (DAB) was used for color development, and hematoxylin was used for nuclear counterstaining. Imaging was performed using a microscope, and quantitative analysis was conducted in ImageJ. A threshold was set to extract DAB signals and calculate the area proportion of positive cells. Hematoxylin staining was used to determine the total number of cells, ensuring comprehensive analysis of the entire tissue area. Measurements from multiple fields were averaged to provide representative quantitative data for statistical analysis.

### 4.30 Biochemical Analysis

Blood was collected from the retro-orbital venous plexus. Samples were allowed to clot at room temperature for 2 h and subsequently centrifuged at 3,000 × g for 15 min at 4 °C to separate serum. Serum aliquots were stored at -80 °C until analysis. Hepatic fibrosis markers were quantified by enzyme-linked immunosorbent assay (ELISA). Hepatic injury markers were determined using an automated biochemical analyzer (Chemray 240, Rayto Life and Analytical Sciences, Shenzhen, China).

### 4.31 Data Analysis

All data were analyzed using GraphPad Prism 8.0 and ImageJ 1.8.0 software. Data from three or more independent experiments are presented as mean ± standard deviation (mean ± SD). Data analysis was performed using paired Student's t-test or one-way analysis of variance (ANOVA), with p < 0.05 considered statistically significant. Significance levels are indicated as follows: *p < 0.05, **p < 0.01.

## Supplementary Material

Supplementary figures.

## Figures and Tables

**Table 1:** The primer sequence.

Gene (Human)	Sequence
SLC31A1	
Forward	5'-GGGGATGAGCTATATGGACTCC-3'
Reverse	3'-TCACCAAACCGGAAAACAGTAG-5'
GAPDH	
Forward	5'-CCAACCGCGAGAAGATGA-3'
Reverse	3'-CCAGAGGCGTACAGGGATAG-5'

**Table 2:** Antibody list.

Primary/Secondary antibody	Company	Cat. No	Dilution
DLAT	Proteintech	13426-1-AP	1:1000
Lipoic Acid	Abcam	AB58724	1:1000
LIAS	HUABIO	HA722085	1:1000
SLC7A11	ABclonal	A13685SP	1:1000
GPX4	Proteintech	30388-1-AP	1:1000
ACSL4	Proteintech	22401-1-AP	1:1000
ATP7B	Proteintech	19786-1-AP	1:1000
RAB1B	Proteintech	17824-1-AP	1:1000
HSP70	Biodragon	RM8223	1:1000
SLC31A1	ABclonal	A10109	1:1000
RAB10	zenbio	R25515	1:1000
VDAC1	Proteintech	10866-1-AP	1:1000
USP11	Proteintech	10244-1-AP	1:1000
KAT2A	Proteintech	28390-1-AP	1:1000
Caveolin1	Proteintech	16447-1-AP	1:1000
Caveolin2	Proteintech	28358-1-AP	1:1000
Aconitase 2	Bioss	Bsm-61944R	1:1000
POLD1	Proteintech	15646-1-AP	1:1000
NDUFB8	Proteintech	14794-1-AP	1:1000
Succinyllysine	Ptm-biolab	PTM-419	1:1000
Ubiquitin	Proteintech	10201-2-AP	1:1000
FDX1	Proteintech	12592-1-AP	1:1000
α-SMA	Proteintech	14395-1-AP	1:1000
Collagen I	Proteintech	67288-1-Ig	1:1000
CD9	zenbio	R380441	1:1000
TSG101	zenbio	R25999	1:1000
GAPDH	Promabio	P04406	1:2000
Goat anti rabbit IgG H&L (HRP)	ABclonal	AS014	1:5000
Goat anti mouse IgG H&L (HRP)	ABclonal	AS003	1:5000

**Table 3:** Sequence information.

Gene name	Sequence
RAB1B	CACGUACACAGAGAGCUACAU
SLC31A1	GCGUAAGUCACAAGUCAGCAU
VDAC1	GCUUGGUCUAGGACUGGAAUU
