# Supplementary material for: DHA Targets RAB1B to Mediate the Driving Force Transmission of Copper Ion Vesicular Endocytosis Under Copper Overload for Promoting Cuproptosis and Ferroptosis in Hepatic Stellate Cells
Source: Int J Biol Sci. 2026 Jul 22;22(13):6914–44. doi: 10.7150/ijbs.134520 (PMC13540460; doi:10.7150/ijbs.134520)
Supplement: Supplementary file 1 [file ijbsv22p6914s1.pdf]

**Supplementary Figure 1**

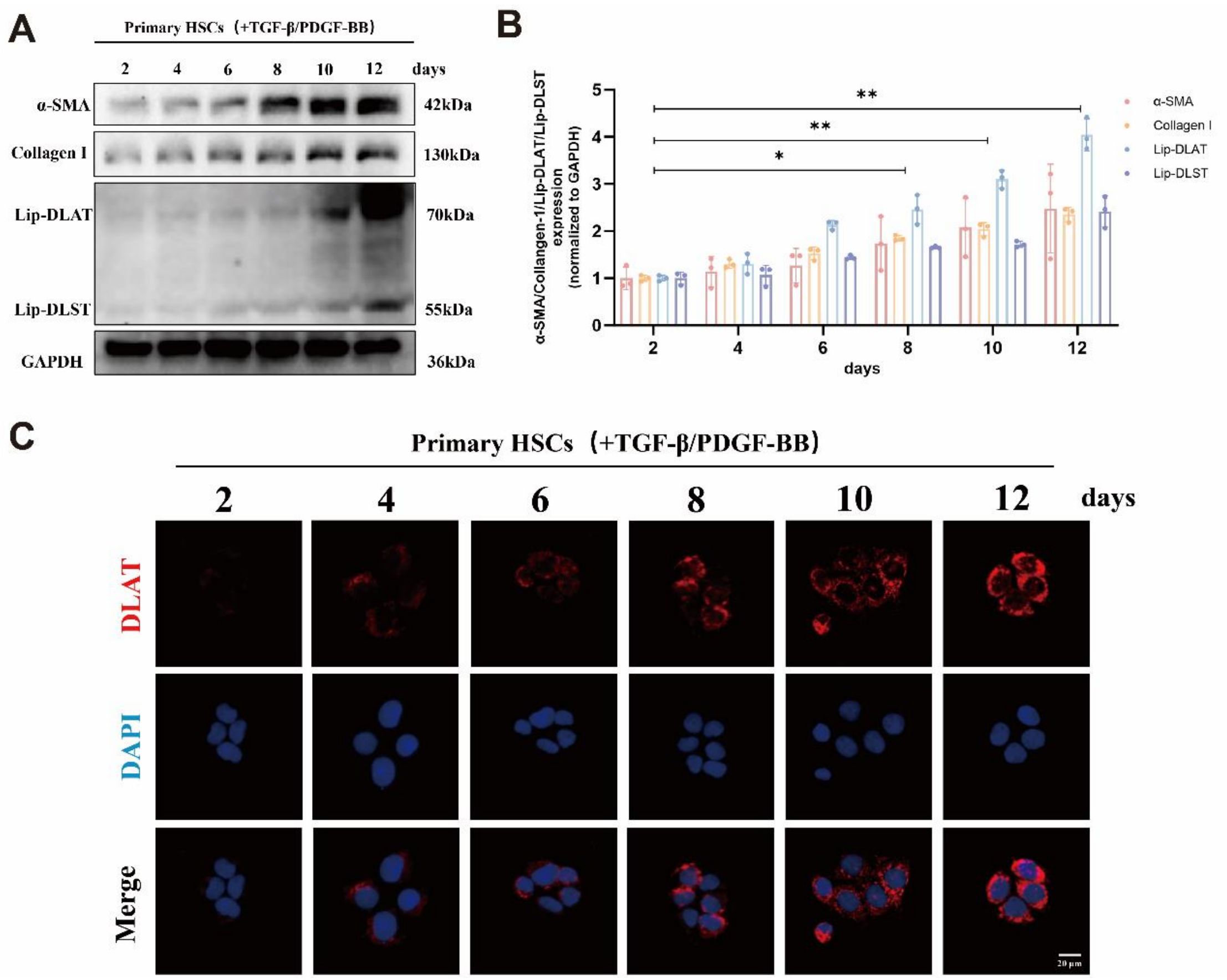


(A-B) Western Blot analysis and quantitative densitometry of Lip-DLAT, Lip-DLST, α-SMA, and Collagen I protein levels in TGF-β/PDGF-BB-activated primary HSCs or the indicated time periods (0-12 days) (n = 3).

(C) Immunofluorescence staining was performed to detect DLAT expression in TGF-β/PDGF-BB-activated primary HSCs.

**Supplementary Figure 2**

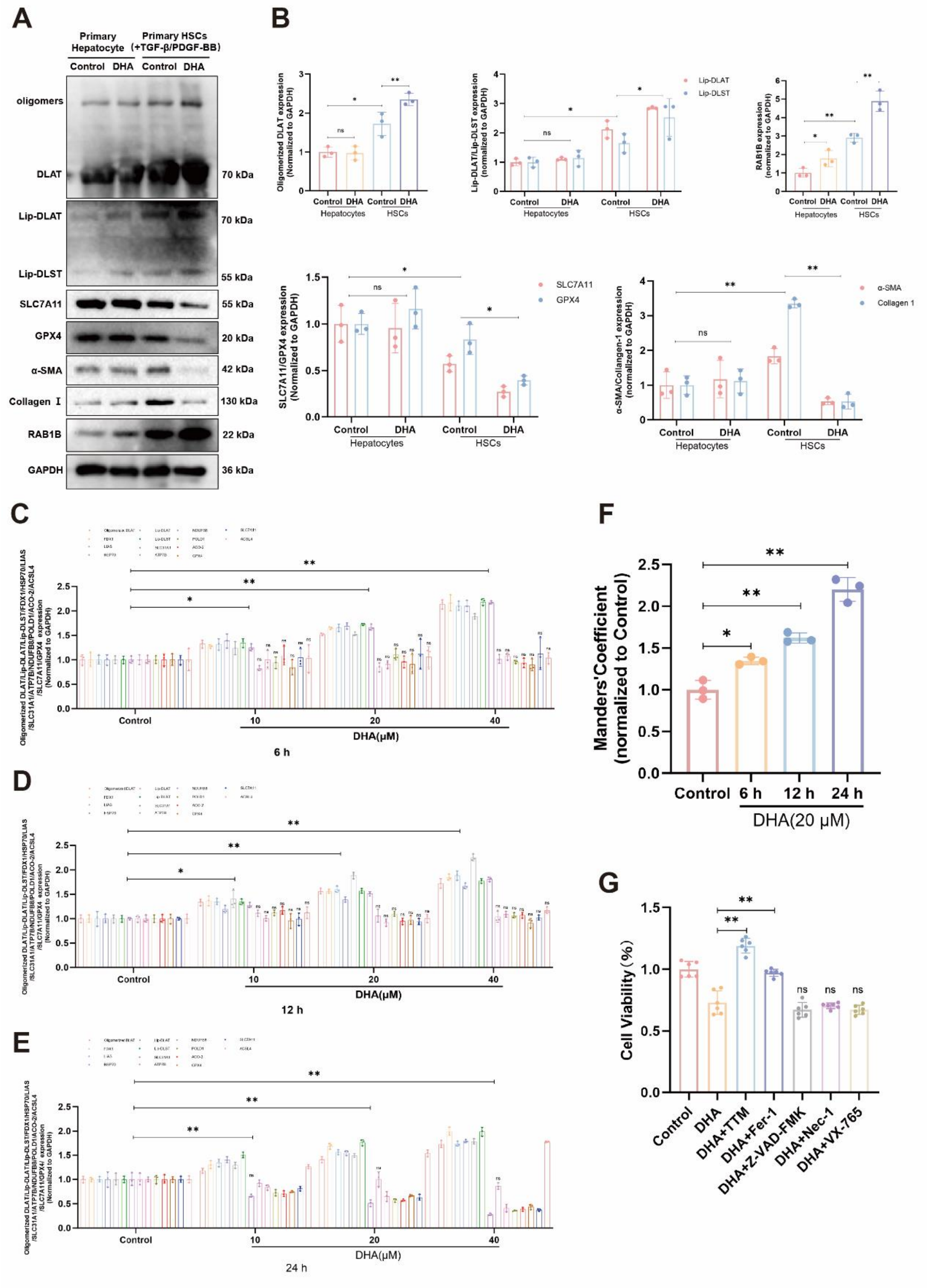


(A-B) Western Blot analysis and quantitative densitometry of oligomerized DLAT, Lip-DLAT, Lip-DLST, SLC7A11, GPX4, α-SMA, Collagen I and RAB1B protein levels in primary hepatocytes and TGF-β/PDGF-BB-activated primary HSCs under control and DHA (20 μM) treatment for 24 hours (n = 3).

(C-E) Quantification of protein levels of DLAT oligomerization, FDX1, LIAS, HSP70,

Lip-DLAT, Lip-DLST, SLC31A1, ATP7B, GPX4, SLC7A11, ACSL4, NDUFB8, POLD1, ACO-2 in LX2 cells at different time points (6 h, 12 h, 24 h) following DHA (0-40 μM) treatment (n = 3).

(F) Quantification of the co-localization of copper and iron ions with mitochondria in LX2 cells at different time points (6 h, 12 h, 24 h) following DHA (20 μM) treatment (n=3).

(G) The effect of different cell death inhibitors on LX2 cell viability was evaluated using the CCK-8 assay (n = 6).

**Supplementary Figure 3**

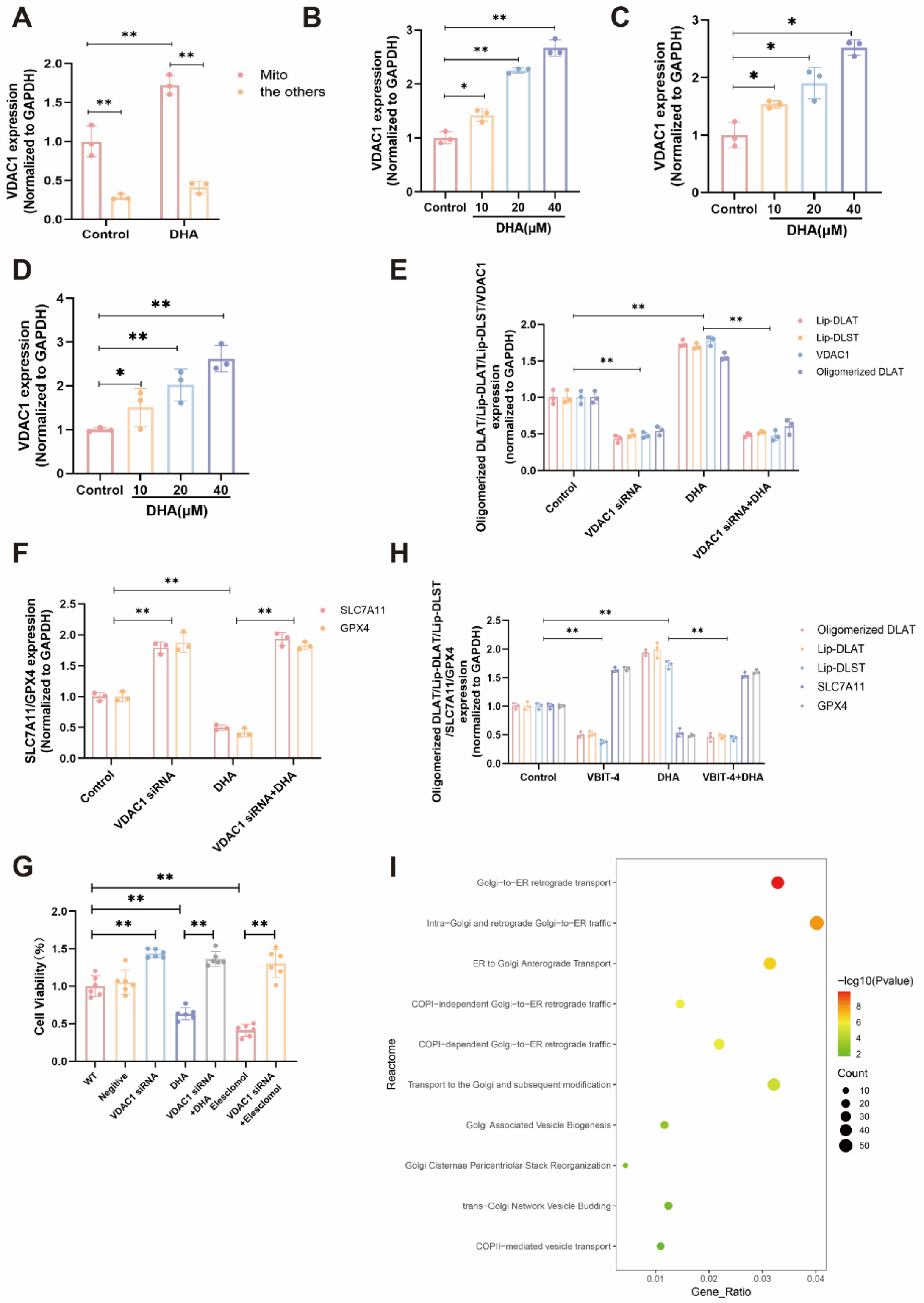


(A) Quantification of VDAC1 protein levels in mitochondria and other cellular structures in LX2 cells following DHA (20 μM) treatment for 24 h (n = 3).
(B-D) Quantification of VDAC1 protein levels in LX2 cells at different time points (6 h, 12 h, 24 h) following DHA (20 μM) treatment (n = 3).
(E-F) Quantification of protein levels of DLAT oligomerization, Lip-DLAT, Lip-

DLST, VDAC1, SLC7A11, and GPX4 in LX2 cells after VDAC1 knockdown by siRNA and DHA (20 μM) treatment for 24 h (n = 3).

(G) CCK-8 assay to measure cell viability of LX2 cells treated with DHA (20 μM) or Elesclomol-Cu (10 nM) for 24 h, along with VDAC1 siRNA or negative control siRNA transfection (n=5).

(H) Quantification of protein levels of DLAT oligomerization, Lip-DLAT, Lip-DLST, SLC7A11, and GPX4 in LX2 cells after combined treatment with VBIT-4 (5 μM) and DHA (20 μM) for 24 h.

(I) Co-IP/MS identification of VDAC1-associated protein interaction network in LX2 cells, followed by Reactome pathway analysis (n=3).

Data are presented as mean ± SD, with p-values calculated using one-way ANOVA. ns, not significant; $*p < 0.05$, $**p < 0.01$.

**Supplementary Figure 4**

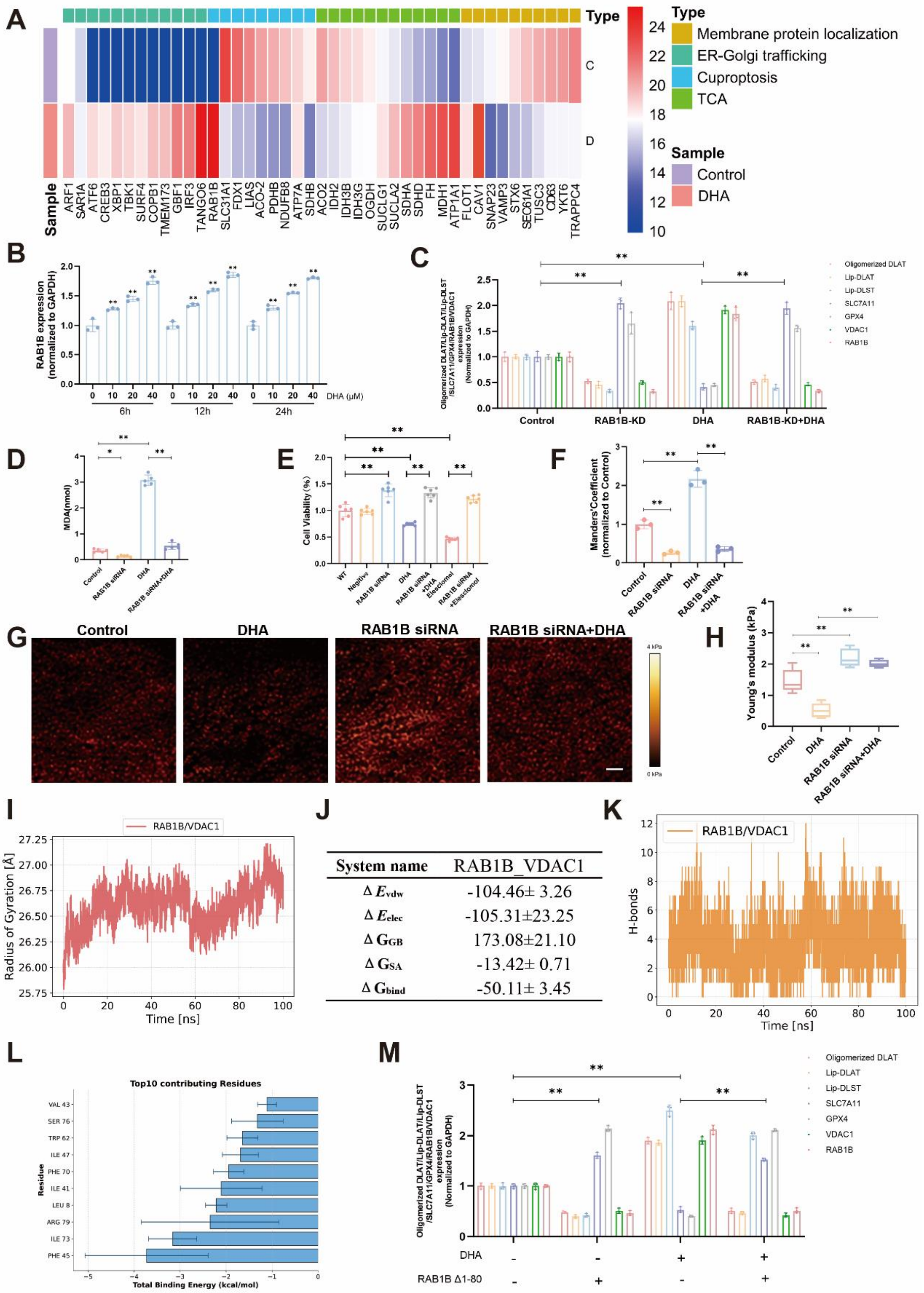


(A) Heatmap of protein expression profiles in LX2 cells after DHA (20 μM) treatment for 24 h. The samples include the control group and DHA group.

(B) Quantification of RAB1B protein levels in LX2 cells after DHA (0-40 μM) treatment at different time points (6 h, 12 h, 24 h) (n = 3).

(C) Quantification of protein levels of DLAT oligomerization, Lip-DLAT, Lip-DLST,

VDAC1, SLC7A11, GPX4, RAB1B in LX2 cells following RAB1B knockdown by siRNA and DHA (20 μM) treatment for 24 h (n = 3).
(D) Measurement of MDA levels in LX2 cells following RAB1B knockdown by siRNA and DHA (20 μM) treatment for 24 h (n = 3).
(E) CCK-8 assay to measure cell viability of LX2 cells treated with DHA (20 μM) or Elesclomol-Cu (10 nM) for 24 h, along with RAB1B siRNA or negative control siRNA transfection (n = 5).
(F) Quantification of VDAC1 and RAB1B co-localization levels in LX2 cells following RAB1B knockdown by siRNA and DHA (20 μM) treatment for 24 h (n = 3).
(G) Atomic force microscopy was used to measure cell stiffness of LX2 cells following RAB1B knockdown by siRNA and DHA (20 μM) treatment for 24 h.
(H) Young's modulus represents cell stiffness.
(I) Radius of gyration (RoG) changes over simulation time for the RAB1B-VDAC1 complex, as predicted by molecular dynamics simulation.
(J) Binding free energies and energy components predicted by MM/GBSA (kcal/mol).
(K) Changes in the number of hydrogen bonds between RAB1B and VDAC1 during molecular dynamics simulation.
(L) Top 10 amino acids contributing to binding on the RAB1B protein.
(M) Quantification of protein levels of DLAT oligomerization, Lip-DLAT, Lip-DLST, VDAC1, SLC7A11, GPX4, RAB1B in LX2 cells treated with DHA (20 μM) and transfected with mutant RAB1B lacking the N-terminal 1-80 aa domain for 24 h (n = 3).
Data are presented as mean ± SD, with p-values calculated using one-way ANOVA. ns, not significant; *p < 0.05, **p < 0.01.

## Supplementary Figure 5

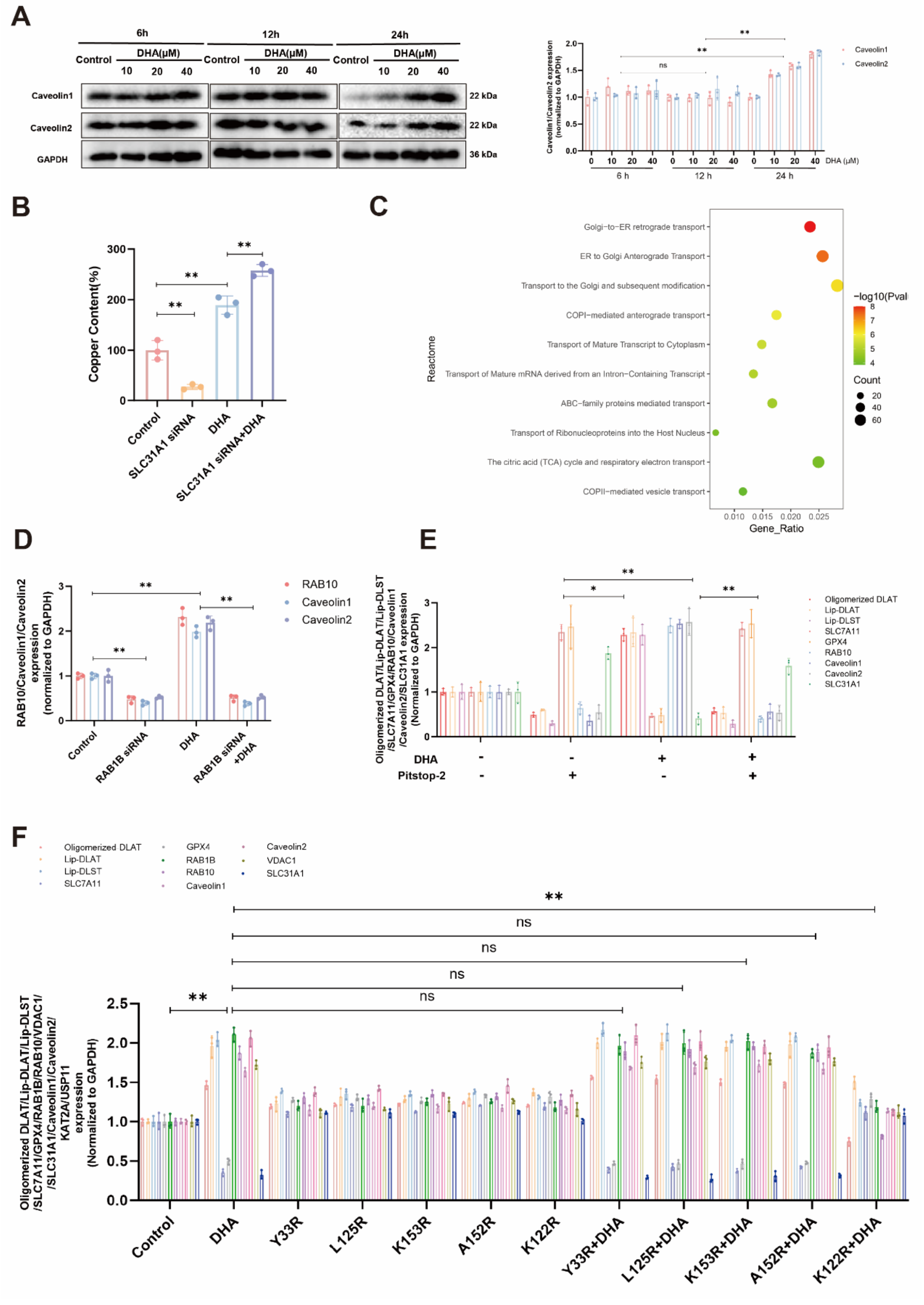


(A) Western blot analysis was used to examine the expression levels of Caveolin1 and Caveolin2 in LX2 cells treated with DHA (0-40 μM) for 6 h, 12 h, and 24 h. Protein expression levels were quantified using grayscale analysis (n = 3).

(B) ICP-MS measurement of copper ion levels in LX2 cells treated with DHA (20 μM) for 24 h and transfected with SLC31A1 siRNA or negative control siRNA (n = 3).

(C) Identification of the protein interaction network associated with copper ion vesicles using Co-IP-MS, followed by Reactome analysis.
(D) Grayscale analysis was performed to quantify the protein expression levels of RAB10, Caveolin1, and Caveolin2 in LX2 cells treated with DHA (20 μM) for 24 h and transfected with RAB1B siRNA or negative control siRNA (n = 3).
(E) Quantification of SLC31A1, GPX4, SLC7A11, RAB10, Caveolin1, Caveolin2, Lip-DLST, Lip-DLAT, and oligomerization of DLAT protein levels in LX2 cells after 24 h of treatment with DHA (20 μM) alone or in combination with Pitstop-2 (20 μM) (n = 3).
(F) Quantification of RAB1B, SLC31A1, VDAC1, GPX4, SLC7A11, RAB10, Caveolin1, Caveolin2, Lip-DLST, Lip-DLAT, and oligomerization of DLAT protein levels in LX2 cells transfected with wild-type or mutant RAB1B (Y33R, L125R, K153R, A152R, and K122R) plasmids for 48 h prior to DHA (20 μM) treatment for 24 h (n = 3).
Data are presented as mean ± SD, with p-values calculated using one-way ANOVA. ns, not significant; $*p < 0.05$, $**p < 0.01$.

**Supplementary Figure 6**

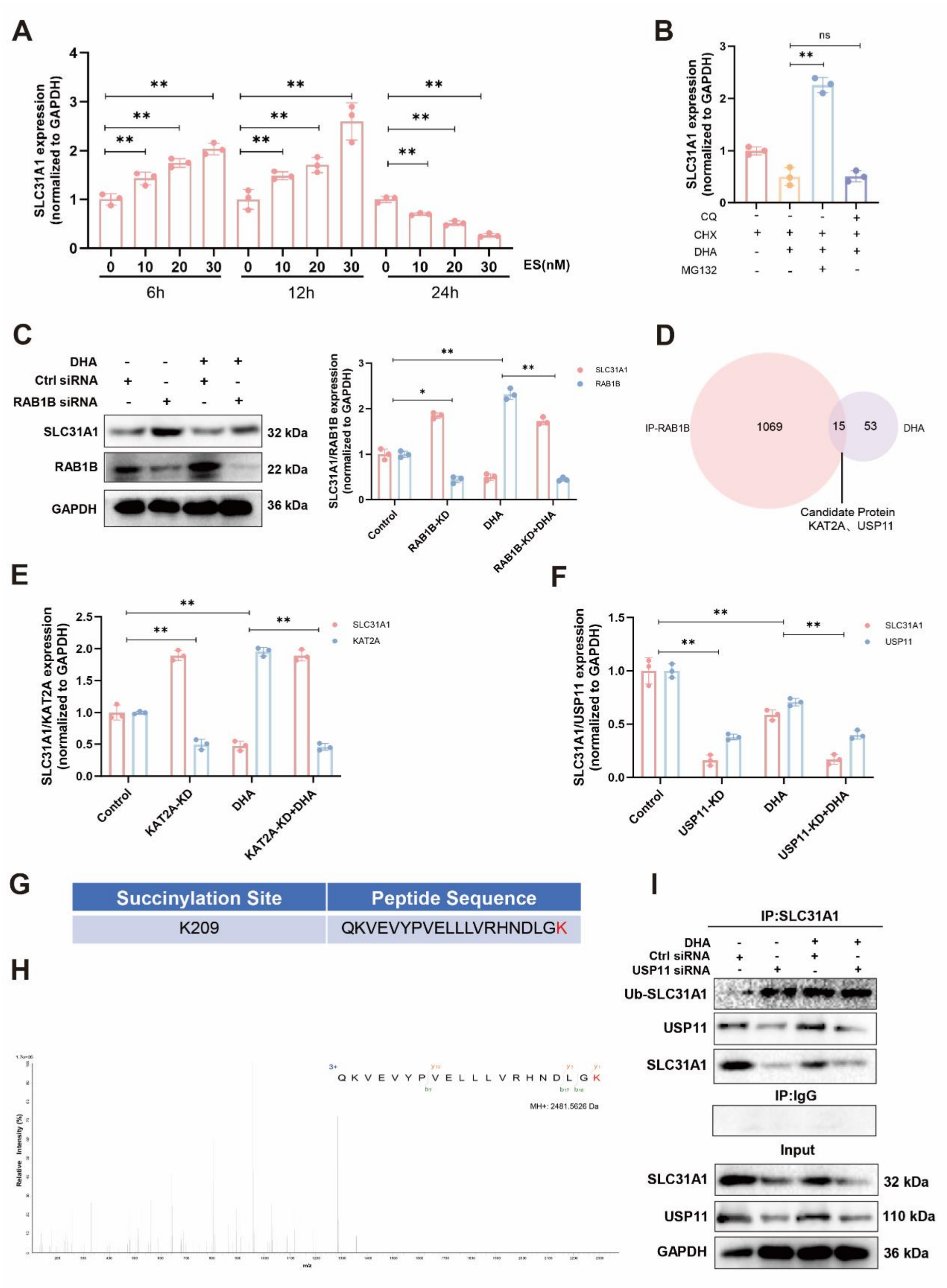


(A) Quantification of SLC31A1 protein levels in LX2 cells after 0-24 h of treatment with Elesclomol-Cu (0-30 nM) (n = 3).

(B) Quantification of SLC31A1 protein levels in LX2 cells after 24 hours of treatment with DHA (20 μM), CHX (20 μg/ml), MG132 (10 μM), CQ (5 μM), either alone or in combination (n = 3).

(C) Western blot was performed to detect the expression level of SLC31A1 protein in LX2 cells treated with DHA (20 μM) for 24 h following transfection with RAB1B siRNA or negative control siRNA, and quantified by densitometric analysis (n = 3).
(D) Intersection analysis of differential proteins.
(E) Grayscale analysis was performed to quantify the protein expression levels of SLC31A1 in LX2 cells treated with DHA (20 μM) for 24 h and transfected with KAT2A siRNA or negative control siRNA (n = 3).
(F) Grayscale analysis was performed to quantify the protein expression levels of SLC31A1 in LX2 cells treated with DHA (20 μM) for 24 hours and transfected with USP11 siRNA or negative control siRNA (n = 3).
(G) Succinylation modification sites and sequences on USP11.
(H) Identification of USP11 succinylation sites by mass spectrometry.
(I) Co-IP was performed to examine the interaction between USP11 and SLC31A1 and to detect the ubiquitination level of SLC31A1 protein in LX2 cells transfected with USP11 siRNA or negative control siRNA and treated with DHA (20 μM) for 24 h (n = 3).
Data are presented as mean ± SD, with p-values calculated using one-way ANOVA. ns, not significant; *$p < 0.05$, **$p < 0.01$.

## Supplementary Figure 7

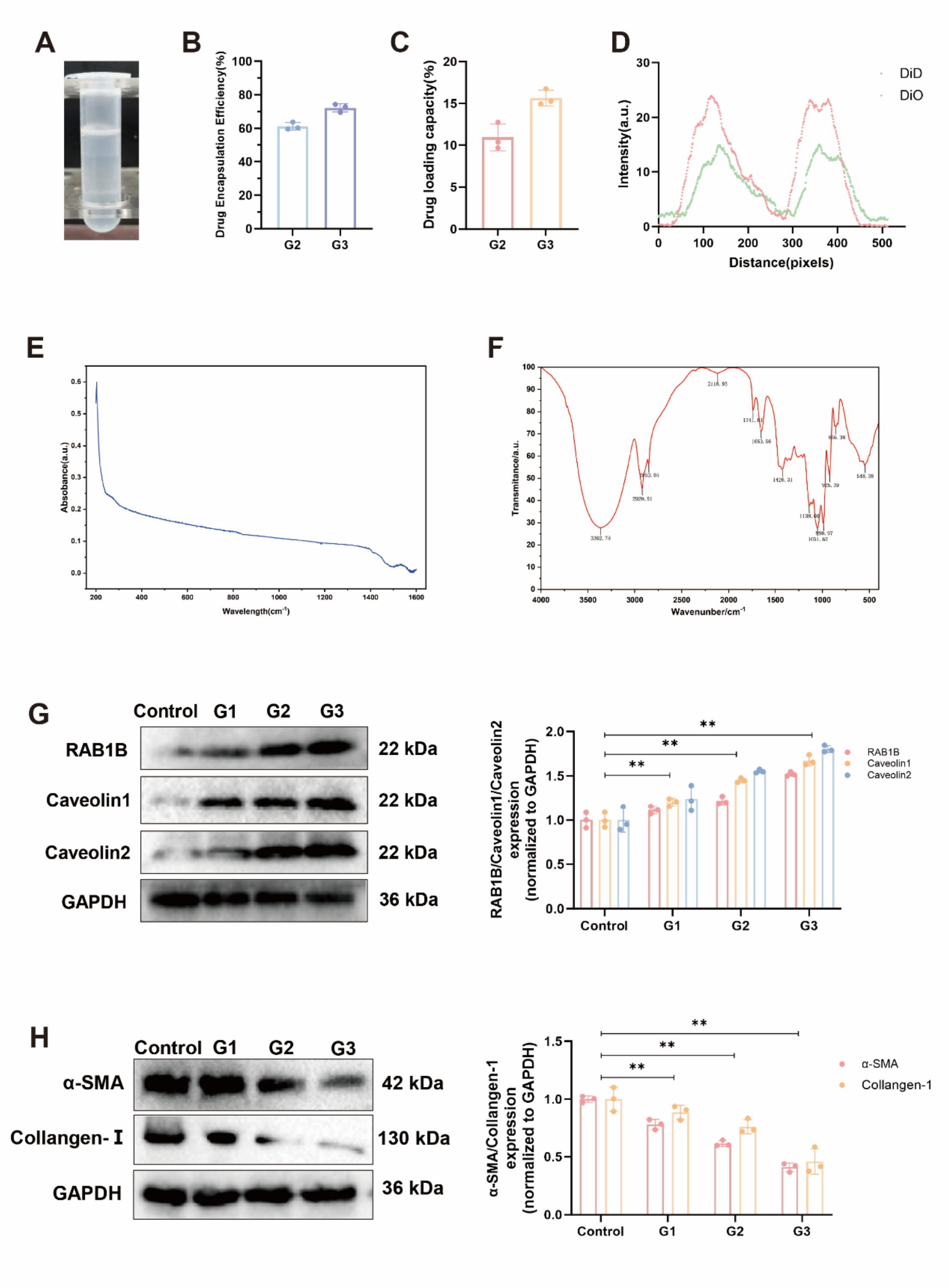


(A) Visual observation of DHA@HSCM-Lipo in physiological saline.

(B) Determination of DHA encapsulation efficiency (n=3).

(C) Determination of DHA drug loading efficiency (n=3).

(D) Fluorescence colocalization analysis of liposomes (labeled with DiD, red fluorescence) and HSC membranes (labeled with DiO, green fluorescence).

(E) UV-Visible spectroscopy analysis of DHA@HSCM-Lipo.
(F) FTIR spectroscopy of DHA@HSCM-Lipo.
(G) Western blot analysis of RAB1B, Caveolin1, and Caveolin2 protein expression in LX2 cells treated with DHA, DHA@Lipo, or DHA@HSCM-Lipo for 24 h. Protein levels were quantified using grayscale analysis ($n = 3$).
(H) Western blot analysis of α-SMA and Collagen I protein expression in LX2 cells treated with DHA, DHA@Lipo, or DHA@HSCM-Lipo for 24 h. Protein levels were quantified using grayscale analysis ($n = 3$).
Data are presented as mean ± SD, with p-values calculated using one-way ANOVA. ns, not significant; $^{*}p < 0.05$, $^{**}p < 0.01$.

**Supplementary Figure 8**

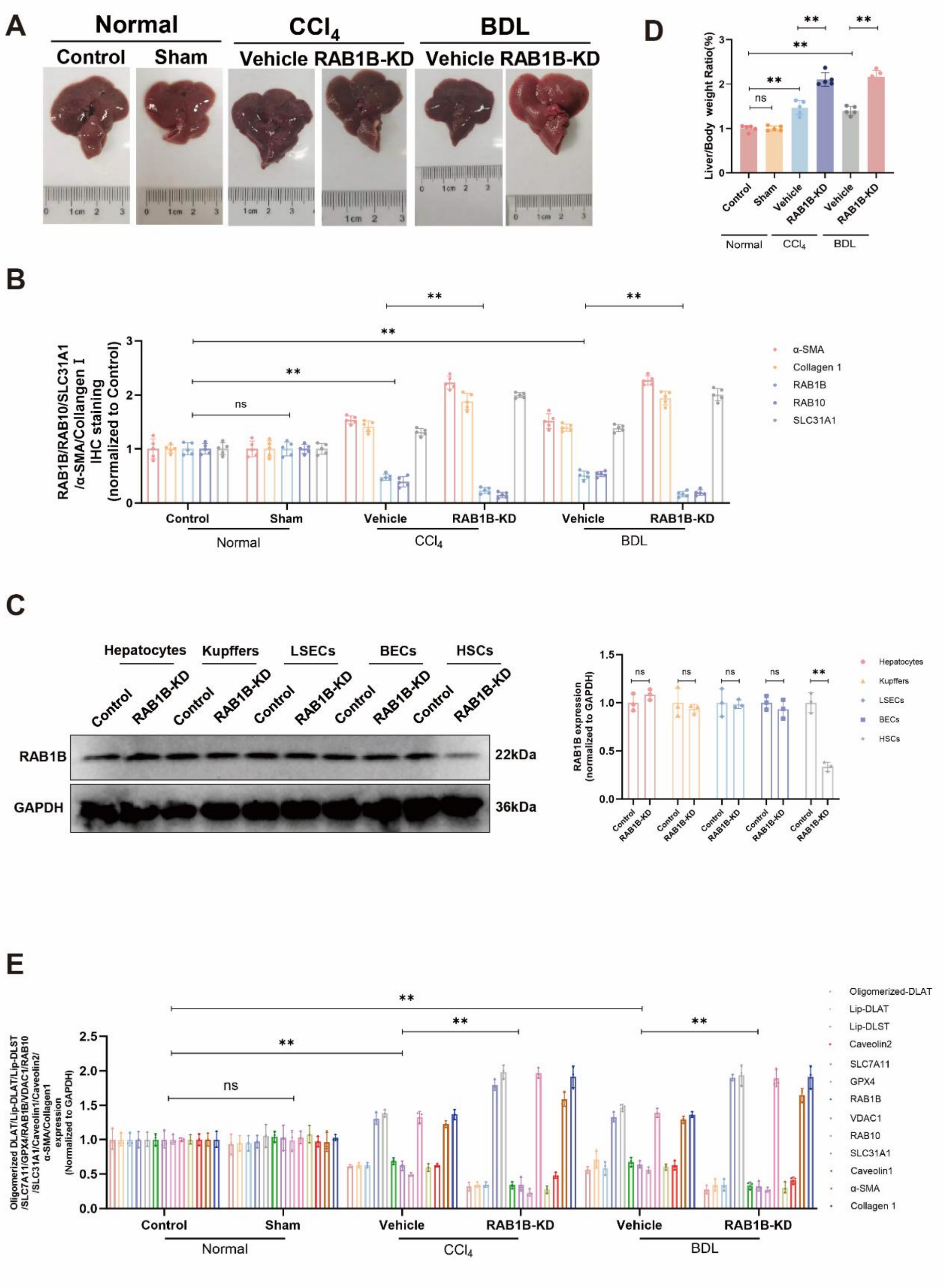


(A) Liver morphology images from normal control mice, sham-operated mice, and $CCl_4$/BDL-induced fibrotic mice (Vehicle and RAB1B-KD groups).

(B) Quantification of α-SMA, Collagen I, RAB1B, SLC31A1, and RAB10 IHC levels in liver tissue from normal controls mice, sham-operated mice, and $CCl_4$/BDL-induced mouse liver fibrosis models (Vehicle and RAB1B-KD groups) (n = 5)

(C) The protein expression levels of RAB1B in hepatocytes, Kupffer cells, LSECs, BECs, and HSCs were detected by Western Blot, followed by quantitative analysis of the band intensities. (n = 3).

(D) Calculation of liver-to-body weight ratio (n = 5).

(E) Quantification of α-SMA, Collagen I, RAB1B, SLC31A1, RAB10, VDAC1, SLC7A11, GPX4, Caveolin1, Caveolin2, Lip-DLST, Lip-DLAT, and oligomerized DLAT protein levels in liver tissue from normal control mice, sham-operated mice, and $CCl_4$/BDL-induced mouse liver fibrosis models (Vehicle and RAB1B-KD groups) (n = 3).

Data are presented as mean ± SD, with p-values calculated using one-way ANOVA. ns, not significant; *$p < 0.05$, **$p < 0.01$.

## Supplementary Figure 9

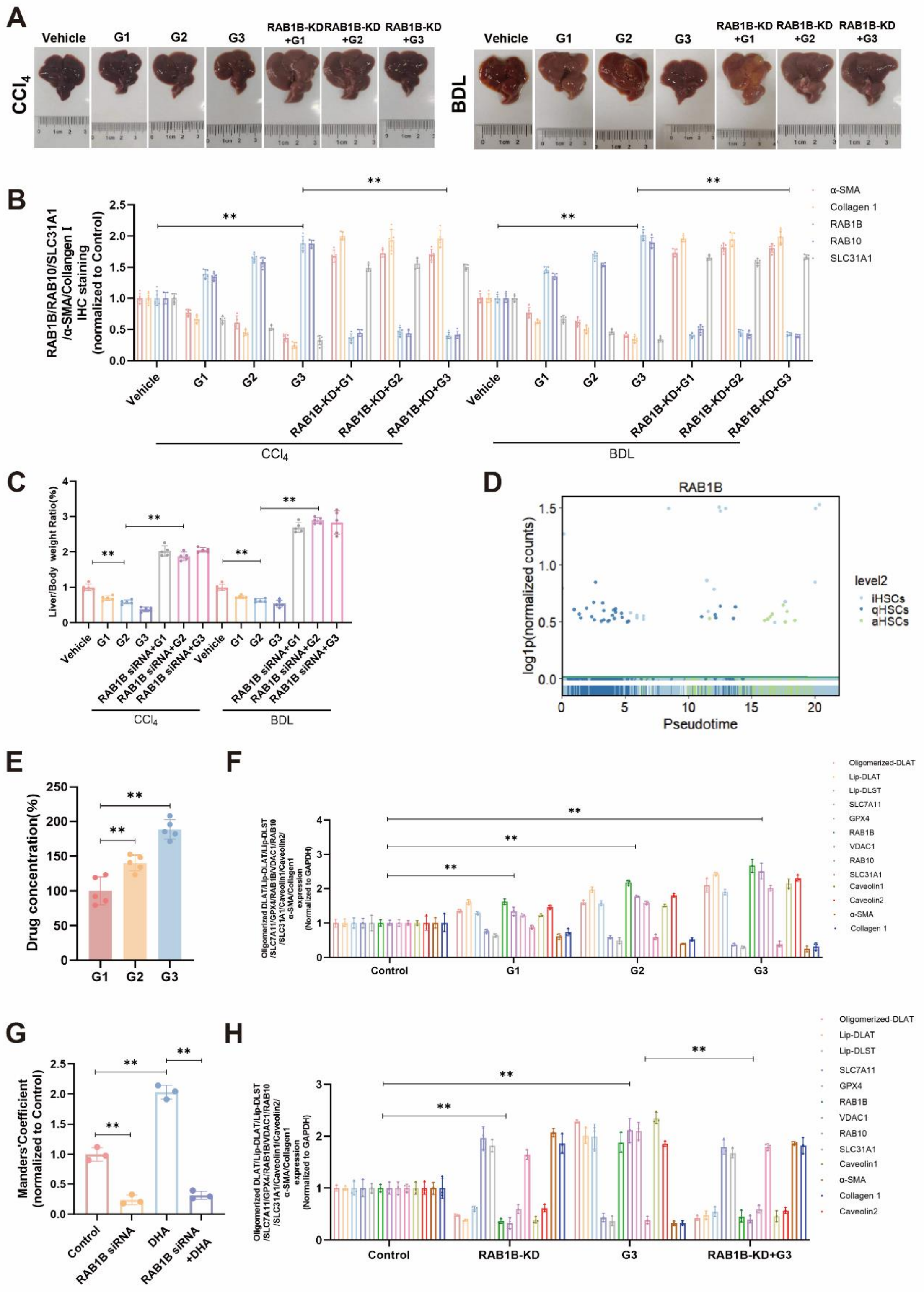

(A) Liver morphology images.

(B) Quantification of α-SMA, Collagen I, RAB1B, RAB10, and SLC31A1 expression by immunohistochemistry in liver tissue from $CCl_4$/BDL-induced mouse liver fibrosis models (n = 5).

(C) Calculation of liver-to-body weight ratio (n = 5).

(D) The expression level of RAB1B along the pseudotime axis in pseudotemporal analysis.
(E) Measurement of in vivo drug accumulation after treatment with DHA, DHA@Lipo, or DHA@HSCM-Lipo.
(F) Quantification of α-SMA, Collagen I, SLC31A1, RAB1B, VDAC1, SLC7A11, GPX4, RAB10, Caveolin1, Caveolin2, Lip-DLST, Lip-DLAT, and oligomerized DLAT protein levels in TGF-β/PDGF-BB-activated primary HSCs treated with DHA, DHA@Lipo, and DHA@HSCM-Lipo ($n = 3$).
(G) Immunofluorescence analysis of copper and iron ion co-localization in TGF-β/PDGF-BB-activated primary HSCs treated with DHA@HSCM-Lipo ($n = 3$).
(H) Quantification of α-SMA, Collagen I, SLC31A1, RAB1B, VDAC1, SLC7A11, GPX4, RAB10, Caveolin1, Caveolin2, Lip-DLST, Lip-DLAT, and oligomerized DLAT protein levels in TGF-β/PDGF-BB-activated primary HSCs with RAB1B knockdown after DHA@HSCM-Lipo treatment ($n = 3$).
Data are presented as mean ± SD, with p-values calculated using one-way ANOVA. ns, not significant; $^{*}p < 0.05$, $^{**}p < 0.01$.
